# Clonally Expanded Virus-Specific CD8 T Cells Acquire Diverse Transcriptional Phenotypes During Acute, Chronic, and Latent Infections

**DOI:** 10.3389/fimmu.2022.782441

**Published:** 2022-02-02

**Authors:** Raphael Kuhn, Ioana Sandu, Andreas Agrafiotis, Kai-Lin Hong, Danielle Shlesinger, Daniel Neimeier, Doron Merkler, Annette Oxenius, Sai T. Reddy, Alexander Yermanos

**Affiliations:** ^1^ Department of Biosystems Science and Engineering, ETH Zurich, Basel, Switzerland; ^2^ Institute of Microbiology, ETH Zurich, Zurich, Switzerland; ^3^ Department of Pathology and Immunology, University of Geneva, Geneva, Switzerland; ^4^ Division of Clinical Pathology, Geneva University Hospital, Geneva, Switzerland

**Keywords:** T cell, single-cell ‘omics’, repertoire, viral, infection

## Abstract

CD8+ T cells play a crucial role in the control and resolution of viral infections and can adopt a wide range of phenotypes and effector functions depending on the inflammatory context and the duration and extent of antigen exposure. Similarly, viral infections can exert diverse selective pressures on populations of clonally related T cells. Technical limitations have nevertheless made it challenging to investigate the relationship between clonal selection and transcriptional phenotypes of virus-specific T cells. We therefore performed single-cell T cell receptor (TCR) repertoire and transcriptome sequencing of virus-specific CD8 T cells in murine models of acute, chronic and latent infection. We observed clear infection-specific populations corresponding to memory, effector, exhausted, and inflationary phenotypes. We further uncovered a mouse-specific and polyclonal T cell response, despite all T cells sharing specificity to a single viral epitope, which was accompanied by stereotypic TCR germline gene usage in all three infection types. Persistent antigen exposure during chronic and latent viral infections resulted in a higher proportion of clonally expanded T cells relative to acute infection. We furthermore observed a relationship between transcriptional heterogeneity and clonal expansion for all three infections, with highly expanded clones having distinct transcriptional phenotypes relative to less expanded clones. Together our work relates clonal selection to gene expression in the context of viral infection and further provides a dataset and accompanying software for the immunological community.

## Introduction

T cells adopt a wide range of phenotypes and effector functions to orchestrate host-defense against infection. Viral infections can be loosely divided into acute and persistent (chronic and latent) infections, with influenza and SARS-CoV-2 being examples of the former and human immunodeficiency virus (HIV), cytomegalovirus (CMV), and hepatitis B virus examples of the later. Murine models of acute, chronic, and latent viral infections have been used to investigate the diverse phenotypes and functions of CD8+ T cells and have been instrumental in characterizing effector, memory, exhausted, inflationary, and self-renewing T cell populations (1–5).

Laboratory studies with the lymphocytic choriomeningitis virus (LCMV) in mice have revealed that acute infections are characterized by the rapid recruitment and differentiation of virus-specific effector CD8+ T cells that enable viral clearance within days (6). This is in contrast to chronic LCMV infections, where prolonged TCR stimulation results in the upregulation of inhibitory molecules and a decrease in effector capabilities, collectively termed T cell exhaustion (3). Finally, infection with another common mouse virus, murine cytomegalovirus (MCMV), has demonstrated to induce a population of expanded CD8+ T cells that respond to the latent reactivation events characteristic of herpes viruses, collectively termed inflationary T cells (5, 7–9). Reductionist approaches involving transgenic animals have been instrumental to characterize infection-specific T cell phenotypes, as transgenic CD8+ T cells expressing virus-specific TCRs can be transferred into naive hosts and profiled following viral infection (4). While this approach is crucial to remove the possible variability between TCR affinities and avidities, it nevertheless introduces into the host an artificially high number of virus-specific CD8+ T cells expressing the same TCR. Similarly, as thousands of transferred TCR transgenic T cells are introduced into naive mice, it is challenging to relate clonal relationships to the dynamic phenotypes at the single-cell resolution.

While recent studies have leveraged bulk sequencing of the TCR beta (TRB) chain during acute, chronic, and latent murine infections (5, 10, 11), these methodologies are inherently limited by the inability to accurately access clonal expansion and further relate transcriptional profiles to those expanded TRB clones. Recent advances in single-cell immune repertoire sequencing can link the complete TCR beta and alpha sequence (VDJ) to gene expression (GEX) at the single-cell resolution (12–14). This technology has recently demonstrated dynamic clonal and transcriptional profiles for virus-specific CD4+ T cells in the context of acute LCMV infection (15), however, it remains unknown how previously described memory, effector, exhaustion, and inflationary phenotypes of virus-specific CD8+ T cells relate to antigen-driven clonal selection. We therefore performed single-cell TCR repertoire sequencing to investigate how the virus-specific CD8 T cell response varies across acute, chronic and persistent infections, which resulted in infection-specific transcriptional fingerprints. We additionally discovered a largely private and polyclonal T cell response in all three infection models, with chronic and latent infection showing higher levels of clonal expansion. Finally, our results indicate that expanded clones preferentially occupy distinct transcriptional clusters and CD8+ T cell phenotypes across all three infections.

## Results

### Single-Cell Sequencing Recovers Diverse Transcriptional Signatures of Virus-Specific CD8+ T Cells

**Figure 1:**
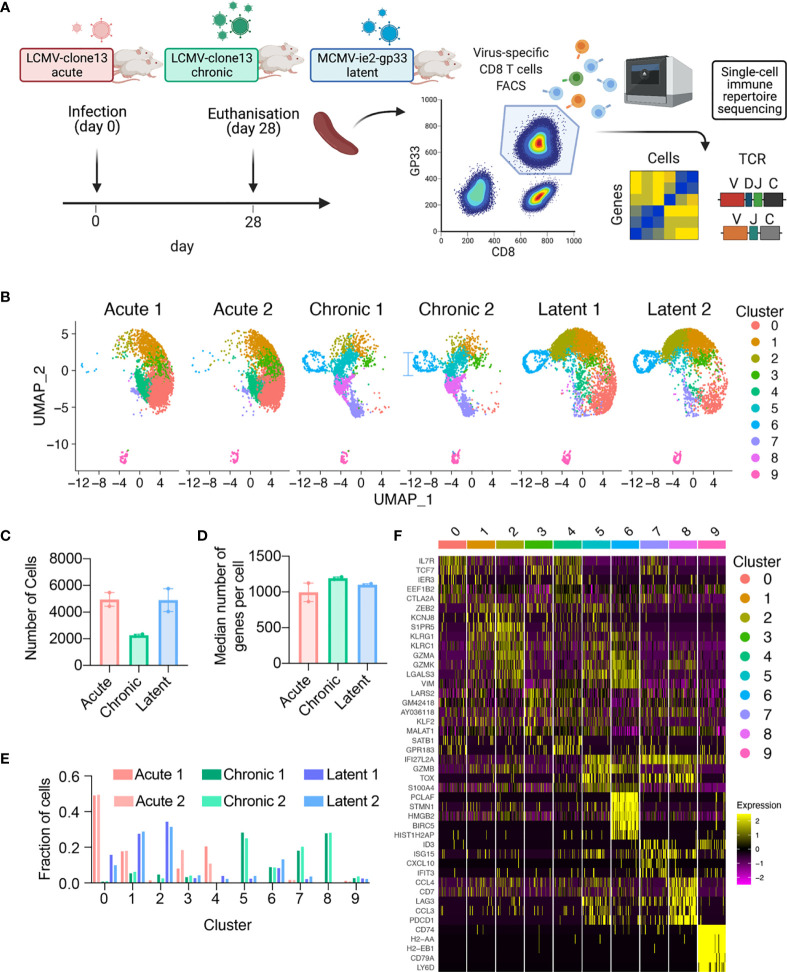
Single-cell immune repertoire sequencing recovers thousands of transcriptomes of virus-specific CD8+ T cells. **(A)** Experimental overview. Created with BioRender.com. **(B)** Uniform manifold approximation projection (UMAP) split by sample. Each point represents a cell and color corresponds to transcriptional clusters. All cells from all samples were integrated in this single UMAP. **(C)** Number of cells with gene expression information for each mouse. **(D)** Median number of genes per cell for each mouse. **(E)** Fraction of cells in a particular transcriptional cluster for each sample. **(F)** Top five significant genes defining each cluster ranked by average log fold change.

To profile the virus-specific CD8 T cell response, we leveraged three previously described models of murine viral infection. In an attempt to maintain initial target cell tropism, the initial distribution of early infection events, and consistency with previous TCR repertoire studies in the context of LCMV infection, acute and chronic LCMV infections were elicited by a low (200 ffu) or high dose (2 x 10^6^) of an identical LCMV clone 13 intravenously (i.v.) as previously performed (4, 10, 16, 17). We additionally induced a latent viral infection *via* i.v. administration of MCMV-*ie2*-gp33 (2x10^5^ pfu) to obtain a stem-like T cell subset comparable to a previously described population of *Tcf7*-expressing cells that sustain the inflationary response during latent infection (5). An advantage of using these three viruses is that they all contain the gp_33-42_ (GP33) viral peptide epitope, which enables isolation of endogenous virus-specific CD8+ T cells using MHC-tetramers. While the GP33 peptide is naturally encoded in the LCMV genome, it has been engineered into the *ie2* gene locus of MCMV and gives rise to a population of virus-specific T cells termed inflationary T cells (5, 18). We therefore isolated GP33-specific CD8+ T cells from the spleen of mice at 28 dpi, separated into cohorts of either acute LCMV, chronic LCMV, or latent MCMV-*ie2*-gp33 infection. While we intended to include GP33-specific CD8+ T cells following peptide immunization with the GP33 peptide in CpG, we were unable to obtain sufficient numbers of GP33-specific cells 28 dpi and therefore excluded this group for future sequencing (**Supplementary Figure S1A**). The virus-specific CD8+ T cells were then processed for single-cell sequencing of their TCR repertoires and transcriptomes by following the 10X genomics workflow (5’ immune profiling with V(D)J and GEX protocol) (**Figure 1A** and **Supplementary Figure S1A**). Following single-cell sequencing and alignment to the murine reference transcriptome, we recovered GEX information from thousands of virus-specific CD8+ T cells from each mouse (**Figures 1B, C**), with the median number of genes per cell ranging from 866 to 1210 for all mice (**Figure 1D**), accompanied by comparable percentages of mitochondrial genes and sequencing reads for all samples (**Supplementary Figures S1B, S1C**).

Acute, chronic, and latent infections have been reported to have distinct phenotypes of virus-specific CD8+ T cells, corresponding to memory, exhaustion, and inflationary subsets (9, 19–22). Performing unsupervised clustering based on total gene expression [excluding genes relating to TCRs, i.e., V-, D-, J-, and constant region (C) genes] and subsequently visualizing the cells from all mice revealed infection-specific clustering (**Figures 1B, D, E**). Quantifying the proportion of cells in each cluster demonstrated that the transcriptional profiles were highly reproducible across biological replicates (**Figure 1E** and **Supplementary Figures S1D, S1E**), with CD8+ T cells found in distinct clusters for acute LCMV (clusters 0,1,3,4), chronic LCMV (5,6,7,8), and latent MCMV (0,1,2,6) infections. Further unbiased investigation into the most expressed genes per cluster revealed a plethora of genes previously reported in the context of viral infections, such as *Il7r*, *Tcf7*, *Zeb2*, *Klrg1*, *Gzma*, *Gzmb*, *Gzmk*, *Vim*, *Lgals3*, *Tox*, *Lag3*, *Pdc1*, *Id3* (**Figure 1B** and **Supplementary Figures S2, S3, S4**). In the case of chronic and latent infections, we observed one cluster (cluster 7) where CD8 T cells express *Tcf7 and Slamf6*, which is characteristic of previously described stem-like T cells capable of sustaining the effector population during chronic infection and during checkpoint blockage (2, 20, 23). Together, these patterns of gene expression suggested the presence of distinct memory (clusters 0, 3, 4), effector (clusters 1, 2, 5), exhausted (cluster 8), memory-like (cluster 7), proliferative (cluster 6) subsets, in addition to a small population of B cells present (cluster 9) in all samples and CD4+ cells, suggesting minor contamination (**Figure 1F**) and were therefore removed for the remainder of the gene expression analyses. We observed considerable overlap of cells from acute LCMV and MCMV-*ie2*-gp33 in the largest effector-like cluster (cluster 1) and subsequently questioned the extent that these cells could be differentiated by gene expression. Performing differential gene expression analysis between the cells of cluster 1 separated by infection type demonstrated that T cells isolated from MCMV-*ie2*-gp33 had significantly increased expression of granzymes (*Gzmk*, *Gzma*), activation genes (*Ccl5*, *Nkg7*, *Klrg1*), and MHC class I genes (**Supplementary Figure S6** and **Supplementary Tables S7, S8**), even though the cells occupied the same cluster (**Figure 1F**). When comparing the cells in cluster 1 arising from either chronic or latent infection, we observed a significant upregulation of genes involved in T cell exhaustion (**Supplementary Figure S6** and **Supplementary Tables S7, S8**). Finally, as we observed a population of stem-like *Tcf7*-expressing cells in both chronic LCMV and MCMV infection, we questioned whether these cells similarly maintained characteristics of exhaustion and T cell inflation, respectively. Differential gene expression between cells within this cluster demonstrated indeed demonstrated that T cells arising from chronic infection within this cluster maintained relatively higher expression levels of exhaustion-program genes such as *Pdcd1, Tox, and Lag3*, in addition to genes such as *Bcl2, Tigit*, and *Id3* (**Supplementary Figure S6**). This was in contrast to *Tcf7+* cells from latent infection, which maintained relatively higher levels of *Gzmm, Ifngr1*, and *Ccl5* (**Supplementary Figure S6**).

### Differential Gene Expression Analysis, Gene Ontology, and Gene Set Enrichment Confirm Memory, Effector, Exhaustion, and Inflationary T Cells

**Figure 2:**
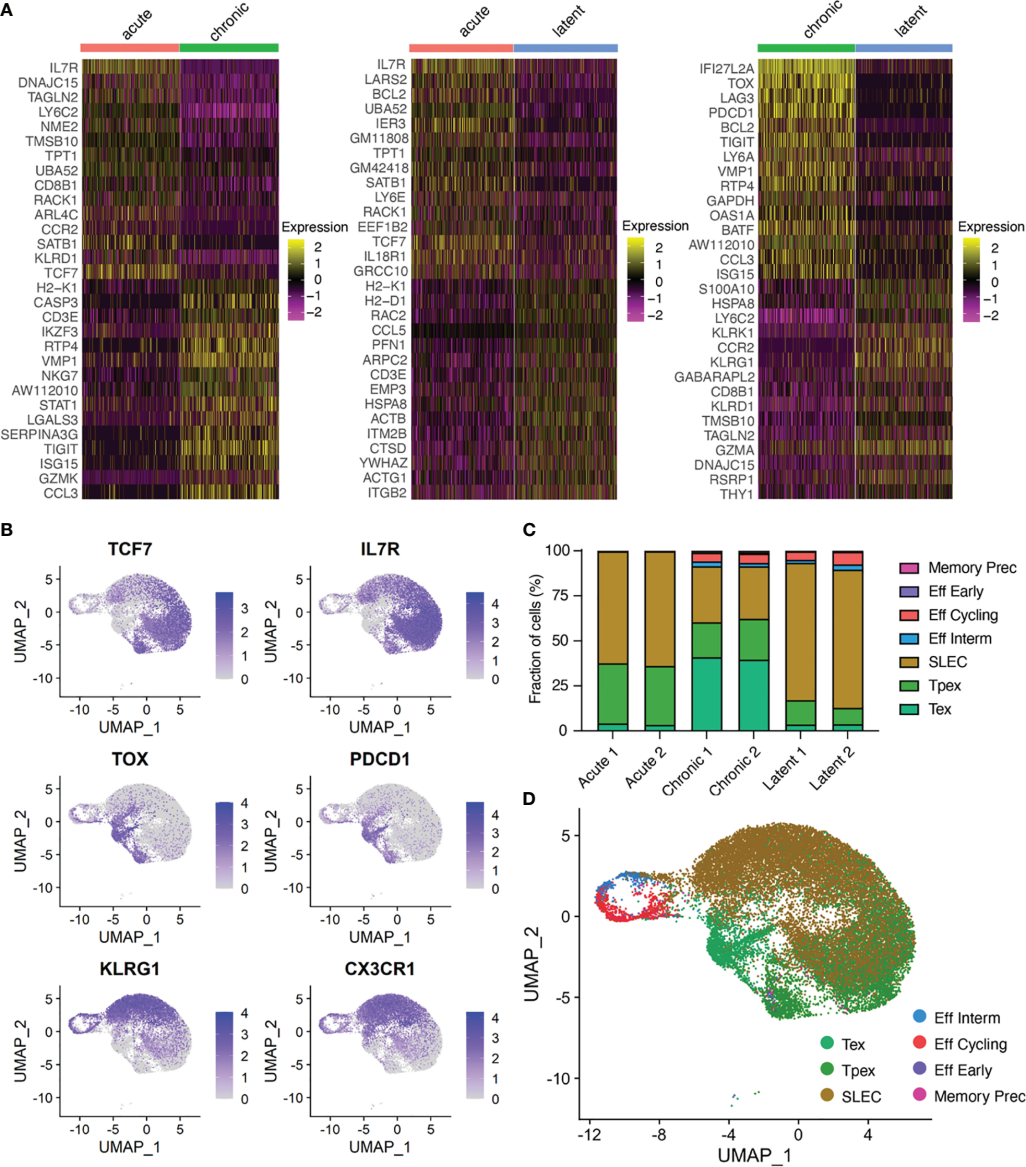
Virus-specific T cells have infection-specific phenotypes. **(A)** Differentially expressed genes between acute and chronic LCMV infection (left), acute and MCMV-ie2-gp33 infections (middle), and chronic and MCMV-ie2-gp33 (right) infections. The upper 15 genes (from top to bottom) correspond to the highest positive average log fold change. Genes 16-30 represent those genes with the lowest average log fold change. All genes displayed have adjusted p values < 0.01. **(B)** Normalized expression for select differentially expressed genes. Each uniform manifold approximation (UMAP) contains cells from all mice. **(C)** Fraction of cells belonging to functional clusters determined by ProjecTILs projected on the acute and chronic viral infection CD8 T cell atlas. **(D)** Uniform manifold approximation projection (UMAP) colored by ProjectTILs functional cluster. Each point represents a cell and color corresponds to transcriptional clusters. All cells from all samples were integrated in this single UMAP.

We next performed differential gene expression and calculated most up and down-regulated genes to determine if infection conditions would further separate transcriptional phenotypes (**Figure 2A**). Genes characteristic of T cell exhaustion were upregulated in the chronic LCMV infection (e.g., *Pdcd1, Tox, Lag3*), whereas genes associated with memory formation and inflationary phenotypes were upregulated in the acute LCMV (e.g., *Il7r*) and latent MCMV (e.g., *Klrg1*) infections (**Figure 2A**, **Supplementary Tables S1–S3**). As many of these genes have been previously described in the context of viral infection, we investigated whether the expression of additional genes commonly used to differentiate populations of CD8+ T cells could further differentiate infection types. Genes such as *Cd8a* and *Cd3e* were expressed ubiquitously across all cells (**Supplementary Figures S2–S4**), whereas exhaustion markers such as *Pdcd1*, *Tim3, Lag3, and Ctla4* were preferentially localized to the cells arising from chronic infection (**Figure 2B** and **Supplementary Figures S2–S4**). We additionally observed a population of cells from chronically infected mice coexpressing *Pdcd1* and *Tcf7* (**Figure 2B** and **Supplementary Figures S2–S4**), which has been described previously as “stem-like”, “memory-like” or “progenitor-exhausted”, and serves to sustain the effector and exhausted population in the context of chronic infection (2, 23, 24). Expression of *Klrg1* was similarly localized to cells arising from the MCMV-ie2-gp33 infection, consistent with the known effector-memory phenotype of inflationary CD8+ T cells (5) (**Figure 2B** and **Supplementary Figures S2–S4**). These findings were consistent when performing gene ontology (GO) and gene set enrichment (GSEA) analyses using the 100 most upregulated genes from our previous differential gene analysis (**Supplementary Figures S6–S8** and **Supplementary Tables S4–S6**).

To better annotate our observed CD8 T cell states, we utilized ProjectTILS (25), a recently develop T cell reference atlas to interpret T cell states. Quantifying the fraction of cells residing in each CD8-specific ProjectTILS-defined subset again highlighted the robust phenotypic differences of T cells arising from the three infection types (**Figures 2C, D** and **Supplementary Figures S9, S10, S11**). Chronic LCMV infection resulted in a clear increase in the exhausted subset, whereas acute and latent infections were largely dominated by short-lived effector cells (SLECs) (**Figure 2C**). When using the generalized T cell reference subsets containing both CD4 and CD8 T cells, however, we observed an increase in naïve-like and early effector-like T cells following acute infection relative to the increased proportion of effector memory cells found following MCMV infection (**Supplementary Figures S10, S11**). Together, this further highlighted distinct transcriptional phenotypes arising from acute, chronic and latent infections.

### Polyclonal but Individualized CD8+ T Cell Clonal Expansion Following Acute, Chronic and Latent Infection

**Figure 3:**
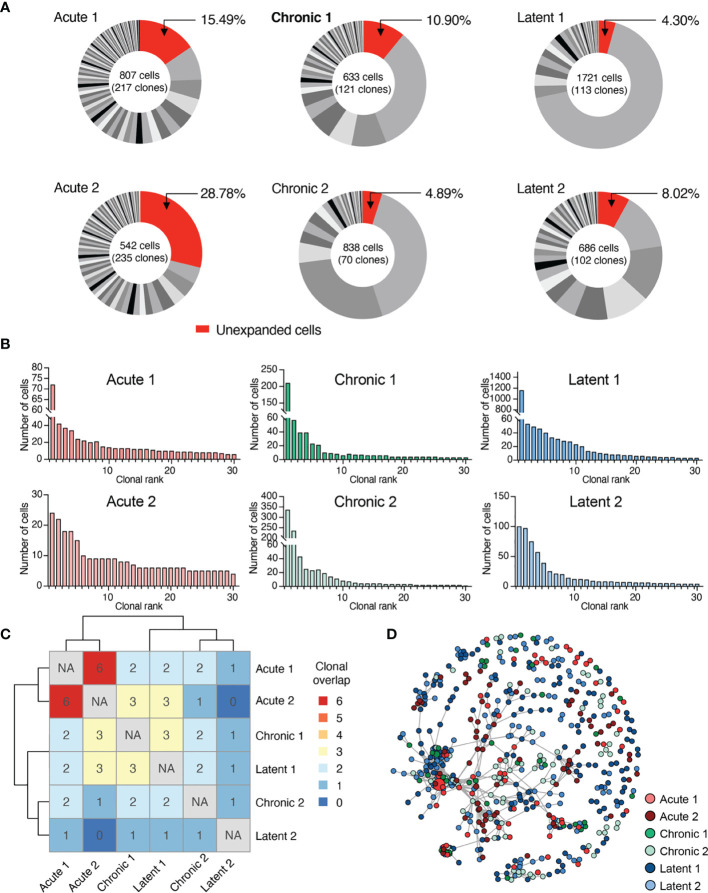
Virus-specific T cells are clonally expanded and personalized. **(A)** Distribution of clonal expansion. Clone was defined by identical CDRb3-CDRa3 nucleotide sequence. Each section corresponds to a unique clone and the size corresponds to the fraction of cells relative to the total repertoire. Lowly expanded clones were those clones supported by only one unique cell barcode. **(B)** Clonal frequency for the top 30 most expanded clones in each repertoire. **(C)** Number of identical clones found between mice. Diagonal comparing identical samples set to not applicable (NA). **(D)** Similarity network of virus-specific CD8+ T cell clones. Nodes represent a unique CDRb3-CDRa3 from each mouse. Edges connect those clones separated by an edit distance of 7 amino acids or less.

After observing the transcriptionally diverse gene expression profiles following the different infection types, we determined if TCR repertoires showed infection-specific features. By restricting our analysis to cells containing only one TRA and one TRB sequence, we obtained information ranging from hundreds to thousands of cells from each mouse (**Figure 3A** and **Supplementary Figure S12A**). Quantifying the number of unique clones [defined by unique complementarity determining region 3 beta (CDRb3) + CDR3 alpha (CDRa3) nucleotide sequence] revealed hundreds of clones for each mouse (**Figure 3B** and **Supplementary Figure S12B**), indicating both a polyclonal GP33-specific repertoire in all three infection conditions and the presence of clonal expansion. Next, we visualized the percentage of the repertoire comprised by each clone, which demonstrated that mice which had received acute LCMV infection (and therefore had already cleared the virus 28 dpi) had a higher fraction of clones supported by only a single cell barcode (**Figure 3A**). Quantifying the Shannon evenness, a commonly used entropy metric that provides a global view of clonal frequencies (26–28), further confirmed the notion that acute LCMV infection resulted in relatively less clonal expansion than the other two infection types, where antigen is still present (**Supplementary Figure S12C**). A closer examination of the 30 most expanded clones from each repertoire (**Figure 3A**) revealed that in many cases individual clones were represented by hundreds of cells, particularly in chronically and latently infected mice, and, in the case of a single latently infected mouse, more than a thousand cells (**Figure 3B**).

We next determined the extent of clonal convergence in the GP33-specific repertoire by quantifying the number of identical TCRs found in each mouse, which revealed minimal overlap detected regardless of infection condition (**Figure 3C**). We observed no prominent relationship between clonal expansion and clonal overlap, as performing a similar analysis restricted to expanded clones (clones supported by two or more cells), including the 10 most expanded clones per mouse, did not reveal any substantial overlap (**Supplementary Figures S12D, E**). We subsequently questioned if signs of clonal convergence could be detected by focusing the analysis on clones with similar, but non-identical TCR sequences. We therefore constructed sequence similarity networks based on the edit distance of the CDRb3 and CDRa3 sequences. Despite investigating a range of edit distance thresholds, we visually failed to observe any infection-specific clustering (**Figure 3D** and **Supplementary Figure S13A**). Formally quantifying the number of edges shared either within the same or across different infection groups supported our visual observation that infection-specific overlap was not present (**Supplementary Figure S13B**).

### Distinct Germline Gene Usage of GP33-Specific CD8+ T Cells Following Acute, Chronic, and Latent Infection

**Figure 4:**
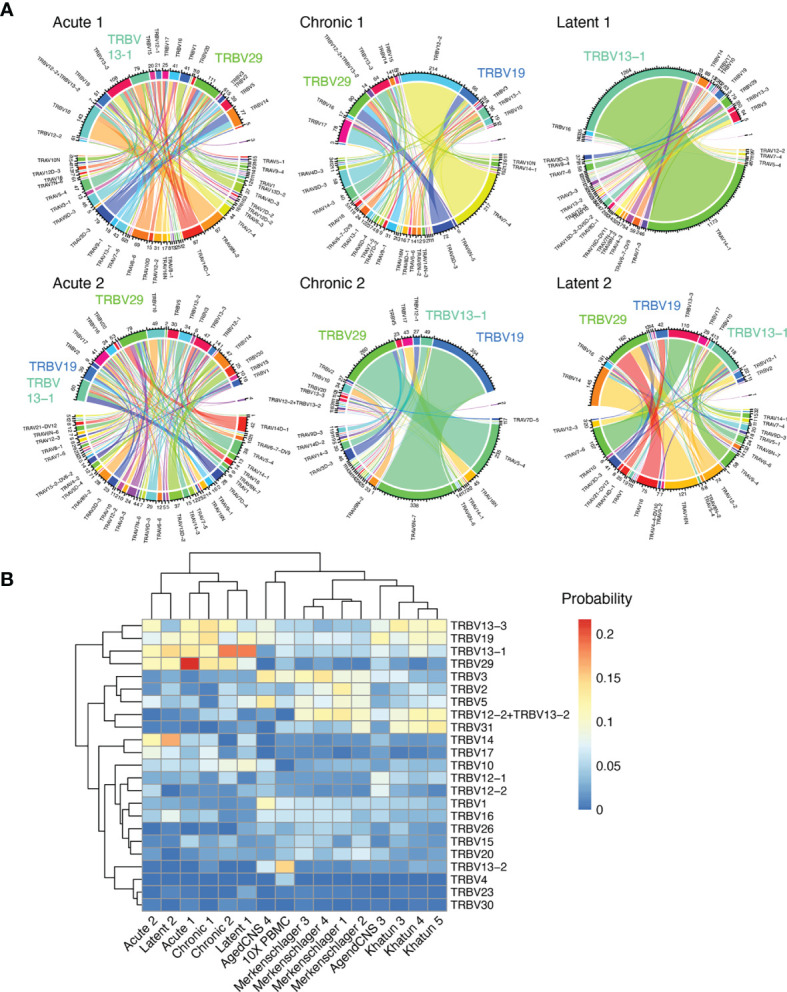
Stereotypic germline gene usage. **(A)** Circos plots depicting the relationship between TRB and TRA V genes. Color corresponds to TRA gene usage. Connections illustrate the number of cells using each particular combination. **(B)** TRB V gene usage compared to other single-cell immune repertoire sequencing datasets containing naive and CD4 T cells.

After observing the low degree of sequence similarity across all mice, we determined if distinct patterns of germline gene usage could be observed in any of the infection conditions, as previous repertoire sequencing experiments investigating virus-specific T cells in the context of LCMV or MCMV infection have demonstrated preferential germline use (5, 10). Leveraging the ability of our single-cell immune repertoire profiling, we could investigate the variable (V) gene usage for both TRB and TRA chains, in addition to quantifying how often certain pairings occurred. Quantifying and visualizing the number of cells using a given germline pairing revealed certain V genes dominated the repertoire across multiple mice in different infection conditions, such as TRBV13-1, TRBV19, and TRBV29 (**Figure 4A** and **Supplementary Figure S14A**). Calculating pairwise correlation coefficients for germline gene usage between all mice demonstrated that TRBV gene usage loosely clustered repertoires by infection type (**Supplementary Figure S14B**), although this effect was not observed when both TRBV and TRAV genes were included into the calculation (**Supplementary Figure S14C**). We lastly questioned whether including repertoires lacking specificity to GP33 could provide contrast for how similar the 6 repertoires were following acute, chronic, and latent infection. We included two naïve datasets from pooled PBMCs from naïve C57BL/6 mice (10x genomics) and CNS-resident CD4 and CD8 T cells from naïve C57BL/6J mice (12). Additionally, GP_66-77_-specific CD4 T cells from C57BL/6 mice following acute LCMV infection (15) and T follicular helper cells from NP17-OVA immunized C57BL/6J mice (29) were included as another source of polyclonal T cell repertoires. Including these publicly available data demonstrated a clear separation between the repertoires of GP33-specific T cells and those T cells arising from other contexts (**Figure 4B** and **Supplementary Figures S15, S16**), further supporting the notion that the GP33-specific repertoire selects for distinct germline gene usage irrespective of the infection condition.

### Transcriptional Heterogeneity Within Expanded Virus-Specific Clones

**Figure 5:**
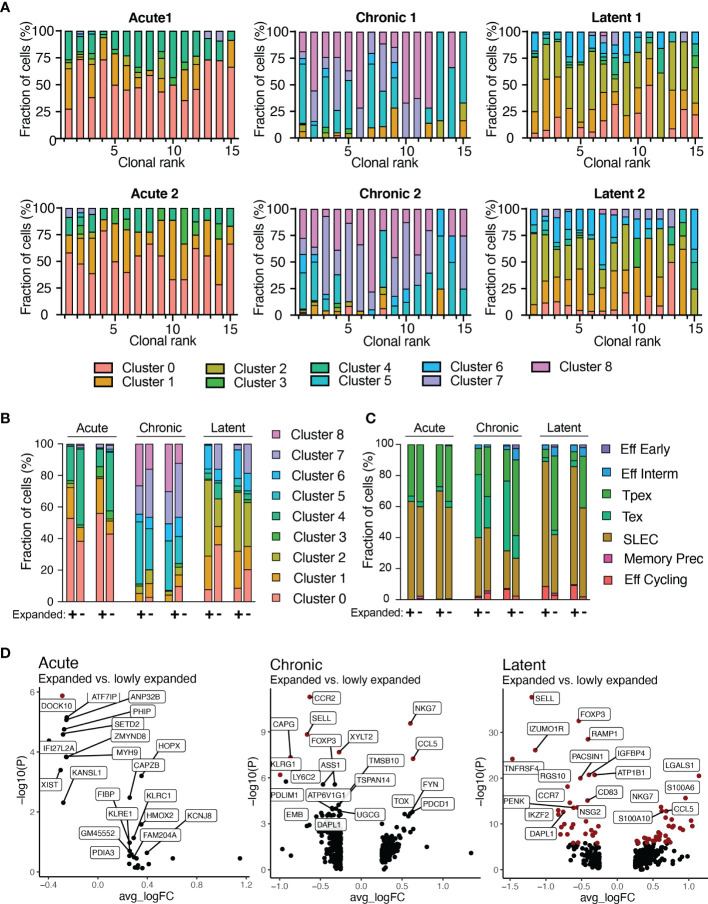
Transcriptional phenotypes relate to clonal expansion. **(A)** Transcriptional cluster membership for the top 15 most expanded clones for each infection group. Clone was defined as identical CDRb3-CDRa3 nucleotide sequence. **(B)** Functional cluster membership determined by ProjectTILs using the acute and chronic viral infection CD8 T cell atlas for expanded (+) and lowly expanded (-) clones for each mouse. Lowly expanded clones were those clones supported by only one unique cell barcode. **(C)** Transcriptional cluster membership for expanded (+) and lowly expanded (-) clones for each mouse. **(D)** Differential gene expression between expanded and lowly expanded cells in the three infection conditions. Points in red indicate significantly differentially expressed genes.

We next integrated TCR sequence with transcriptomes at single-cell resolution. It has been previously demonstrated that highly expanded clones upregulate effector molecules such as *Nkg7*, *Ccl5*, and granzymes (12, 13). Therefore, we first focused our analysis on the 30 most expanded clones for each infection by quantifying the fraction of cells present in each transcriptional cluster (**Figure 5A**). Cells arising from different infection conditions occupied distinct transcriptional states, thereby suggesting transcriptional heterogeneity within the majority of expanded clones (**Figure 5A**). Extending this analysis to all clones, regardless of clonal expansion, would reveal differences between highly and lowly expanded clones. This analysis showed that more expanded clones (thicker lines in **Supplementary Figure S17A**) were predominantly connected to clusters 5 and 2 for chronic and latent infections, respectively, whereas lowly expanded clones (narrower lines) were often connected to clusters 7 and 0, respectively (**Supplementary Figure S17A**). Quantifying the cluster membership demonstrated a trend that those cells with higher expansion levels were located in cluster 1 for acute, 5 for chronic, 2 for latent infection (**Supplementary Figure S17B**), which characteristically expressed *Zeb2*, *Tox*, and *Klrg1*, respectively (**Figures 1B, F** and **Supplementary Figure S2**). Conversely, lowly expanded clones were more often located in clusters 4 for acute and 7 for chronic and latent (**Figure 5B** and **Supplementary Figures S17B, C**), which were characterized by high expression of genes associated with memory phenotypes such as *Id3*, *Sell*, and *Tcf1* (**Figures 1B, F** and **Supplementary Figure S2**). We additionally observed that the stem-like cluster 7 (characterized by *Tcf7* expression) was entirely absent in the highly expanded clones isolated from mice infected with MCMV-*ie2*-gp33. Integrating the ProjectTILS annotations into the clonal expansion data demonstrated similarly support a model in which clonally expanded clones were more likely to reside in certain subsets (e.g., SLEC, effector memory, and exhausted) relative to others (e.g., Pex, naïve-like T cells) (**Figure 5C** and **Supplementary Figure S11**). Calculating the differentially expressed genes revealed that genes such as *Nkg7*, *Lgals1*, *Pdcd1*, and *Ccr2* were significantly upregulated in expanded cells in at least one infection condition and demonstrated consistent trends in expression for all infection groups (**Figure 5D** and **Supplementary Tables S9–S11**). Further inspection into the CDR3 motifs of different transcriptional clusters and the expanded and lowly expanded clones suggested the enrichment of certain residues were enriched in either highly-expanded clones and effector and exhausted clusters (**Supplementary Figure S20**). This was exemplified when visualizing that the lowly-expanded clones from the latent MCMV infection and the *Tcf1*-expressing cluster had increased use of asparagine amino acid in residue nine relative to expanded clones from the latent MCMV infection, which had a preferential use of arginine and higher proportion of clones using glutamine in position 11 similar to expanded cells in effector cluster two (**Supplementary Figure S20**). Taken together, our findings suggest that biophysical properties dictating clonal expansion influence the transcriptional phenotype towards more effector and exhausted phenotypes and away from memory and naïve-like ones.

## Discussion

Here, we used single-cell TCR repertoire and transcriptome sequencing to investigate how T cell clonal selection signatures vary across acute, chronic, and latent viral infection in mice. While the recovered CD8+ T cells shared specificity to a common viral peptide, our results demonstrated infection-specific transcriptional heterogeneity that was maintained across biological replicates. While previous reports have demonstrated that acute, chronic, and latent infections result in T cells with a range of phenotypes and effector functions (2, 3, 5, 30–33), a comparison characterizing whole transcriptomes at the single-cell level has not yet been performed. Our findings showcase the extensive T cell phenotypic diversity and similarly highlight the lack of transcriptional overlap between CD8+ T cell phenotypes from the three models of infection. Consistent with previous results, we could recreate the effector, memory, exhausted, and inflationary expression signatures characteristic of acute, chronic and latent infection using both targeted and unbiased computational analyses. Although utilizing annotation tools such as ProjectTILS revealed that, despite certain subsets being maintained across infection types, distinct transcriptional signatures (e.g. *Gzmb, Ccl5*, NKG7) were nevertheless present in the case of effector cells arising from either low-dose LCMV infection or MCMV-*ie2*-gp33. Whether these differences would remain when comparing inflationary and T cells from the effector-phase of acute infection would be of interest for future work.

Similarly, it remains unknown how comparable the observed transcriptional phenotypes following acute infection would be relative to other models of acute LCMV infection (e,g., higher dose of LCMV Armstrong given either intraperitoneal or intranasal infection), as administration route, strain, and dose influences the virus-specific repertoires and phenotypes. For a comparative analysis between acute and chronic infections, we chose to use an identical viral strain and infection route but at different doses to harmonize the target cell tropism, the extent to which an infected cell responds to the viral infection, and the initial distribution of the early infection events. While using higher dose of LCMV Armstrong to elicit an acute infection would better mirror some infection parameters (e.g. the induction of type 1 IFN, initial viral load and accompanying antigens), it has also been demonstrated that low dose inoculum replicates strongly in the first few days of infection and that the differences in timing and magnitude of the Type 1 IFN response may be marginal (34). Given the consistency in regards to transcriptional phenotypes between our data with previous descriptions of T cells following i.p. and i.v. models of acute infection (4, 16, 20), we would similarly expect to observe similar repertoire features 28 dpi involving the expression profiles of expanded clones, germline gene usage, and CDR3 motifs given that the infection would have been cleared weeks before sacrifice in both models. It is possible, however, that inoculating i.p. with a higher dose of LCMV Armstrong would differentially modulate the clonal expansion profiles of the T cell repertoire at both early time points during the infection and even 28 dpi given the strong influence antigen dose has on the quality and magnitude of the CD8+ T cell response and would warrant future experiments.

The amount and availability of viral antigen is particularly important when comparing latent and chronic infections, as these two infection models have varying viral loads 28 dpi across various organs (5, 6, 35). While it has been shown that the initial dose of MCMV infection dictates the degree of T cell inflation (35), the numbers of inflationary T cells and the fraction of *Tcf7*-expressing cells has been demonstrated to remain stable after approximately three weeks post infection (5). This led us to induce latent infection using an identical strain and dose of MCMV-*ie2*-gp33 as performed in our recent work that had elucidated a population of *Tcf7+* T cells that sustain the inflationary population during MCMV infection (5). Although the aforementioned differences in the antigen load and tissue availability of the GP33-epitope, it was nevertheless interesting that both chronic and latent infection had stem-like *Tcf7+* cells that clustered together and were annotated as the same subset when using ProjectTILS. Interestingly, while lowly-expanded cells in both chronic and latent infections were preferentially located in this *Tcf7+ Il7r+ Slamf6+* cluster (cluster 7) (**Figures 5B, C**), a direct comparison of gene expression between stem-like cells in chronic and latent infection demonstrated that T cells arising from chronic infection maintain relatively higher expression of exhaustion markers (*Pdcd1, Tox, Lag3*) compared to *Tcf7+* T cells from latent infection that maintained higher levels of *Gzmm* and *Ifngr1* (**Supplementary Figure S6**). Future studies comparing the potential of these stem-like *Tcf7+* cells to respond to checkpoint inhibitors would be of interest, as our data suggests that such an intervention would favor cells arising from chronic infection given relatively higher increase in exhaustion markers.

Previous experiments characterizing the endogenous GP33-specific TCR repertoire in the context of LCMV infection have demonstrated varying degrees of polyclonality (10, 11). Leveraging TRB repertoire sequencing, both studies recovered multiple distinct clones, ranging from 40 to hundreds of unique GP33-specific CD8+ T cell clones following chronic and acute LCMV infection. The number of unique clones reported by both studies were comparable to the number of GP33-specific CD8+ T cells found in naive CD8+ T cells of uninfected C57BL/6 mice (36–38). Importantly, both TRB studies demonstrated high clonal overlap between the TCF1+ and TCF1- CD8+ T cell repertoires (10, 11), which together supports a previously proposed model in which TCF1+ CD8+ T cells feed into the TCF1- CD8+ T cell subset (2, 23). Similarly, a high degree of clonal overlap between the TCF1+ and TCF1- repertoires was observed in the context of inflationary T cells following MCMV infection (5). However, as these studies relied upon bulk TRB chain sequencing, relating clonality to gene expression profiles was not possible.

Our single-cell sequencing approach allowed us to relate individual transcriptomes to the TCR repertoire for thousands of cells, thereby providing insight into the relationship between gene expression and clonality. While we could again confirm a polyclonal response through detecting hundreds of unique GP33-specific clones following acute, chronic, and latent infections, we could, for the first time, demonstrate a polyclonal and expanded GP33-specific TCR repertoire at the single-cell resolution. We additionally discovered transcriptional diversity within individual clones that was present in each infection condition. Here, we again observed that clonally expanded T cells are found in both *Tcf1*+ and *Tcf1-* clusters, thereby supporting the previously reported model which implies a clonal relationship between the TCF1+ and TCF1- T cells during chronic viral infection (2). While this hypothesis has been similarly described in the context of cancer (23) and MCMV infection (5), an extensive characterization of this hypothesis at the polyclonal GP33-specific repertoire level was lacking.

In contrast to our previous findings (5, 10), the virus-specific CD8+ TCR repertoires were extremely personalized, with minor clonal overlap between mice. This was true for both expanded and lowly expanded clones, suggesting a stochasticity underlying the selection and expansion of virus-specific clones. The findings presented here may contrast to higher clonal overlap previously reported due to inherent differences in the repertoire sequencing technologies. Specifically, the 10x genomics platform used in this study provides unique molecular identifiers to reduce PCR and sequencing errors and additionally does not rely on multiplex primers, which should improve the accuracy and reduce amplification biases. Although we did not observe a high degree of clonal overlap, we observed that certain germline genes were used more often in TCRs with a common specificity to a single, shared viral epitope. As the naive repertoires of these mice are generated from identical TCR loci, our findings imply that the inflammatory context of distinct infection does not dictate the germline gene selection and accompanying preferential expansion as much as the exact specificity does.

## Methods

### Animal Experiments

All animal experiments were performed in accordance with institutional guidelines and Swiss federal regulations. Experiments were approved by the veterinary office of the canton of Zurich under animal experimentation licenses 115/2017 and ZH058/20. 6-8-week-old female C57BL/6 mice from Janvier were housed under specific-pathogen-free conditions in individually ventilated cages with bedding and nesting material for enrichment. Acute LCMV infections were infected intravenously (i.v.) with 200 focus forming units (ffu) of LCMV clone 13 in the tail vein. Chronic LCMV infections were performed i.v. with 2 x 10^6^ ffu LCMV clone 13. Latent infections were established by injecting 2x10^5^ pfu dose of MCMV-*ie2*-gp33 i.v., which was obtained from Dr. L. Cicin-Sain and contains a functional m157 gene as previously described (18). MCMV viral stocks were propagated on M2-10B4 cells and purified by ultracentrifugation using a 15% sucrose gradient. LCMV clone 13 was produced as previously described (4). Upon sacrifice with CO2 at 28 dpi, spleens were harvested and single-cell suspensions were prepared by mashing the tissue through a 70 uM cell strainer and rinsing with complete RPMI (RPMI-1640 supplemented with 10% fetal bovine serum, 2 mM L-glutamine, 1% penicillin-streptomycin, 1 mM sodium pyruvate, 50 nM beta-mercapthoethanol, 0.1 mM non-essential (glycine, L-alanine, L-asparagine, L-aspartic acid, L-glutamic acid, L-proline, L-serine) amino acids, 20 mM HEPES). The single cell suspension was then incubated with CD8-PE (clone 53-6.7, Biolegend), MHC class 1 tetramer for gp_33-41_ conjugated to APC diluted in FACS buffer (PBS, 2 mmEDTA, 2% FCS) at room temperature for 30 minutes, as previously described (39), and LiveDead nearIR. Tetramer positive cells were isolated *via* flow cytometric sorting (FACSAria with FACSDiva software) and subsequently supplied as input for single-cell immune repertoire sequencing.

### Single-Cell Immune Repertoire Sequencing

Single-cell immune repertoire sequencing was performed as according to the 10x Genomics Chromium Single Cell V(D)J Reagents Kit (CG000166 Rev A) as previously described (40). In brief, single cells for all six samples were simultaneously encapsulated with gel emulsion microdroplets (10x Genomics, 1000006) in droplets using 6 lanes of one Chromium Single Cell A Chip (10x Genomics, 1000009) with a target loading of 13,000 cells per reaction. cDNA amplification was performed using 14 cycles and subsequently split for downstream GEX and VDJ library preparation. GEX libraries were amplified using the Chromium Single Cell 5’ Library Kit (10x Genomics, 1000006). TCR libraries were amplified using the Chromium Single Cell V(D)J Enrichment Kit, Mouse T Cell (10x Genomics, 1000071). Final libraries were pooled and sequenced on the Illumina NovaSeq S1 using a concentration of 1.8 pM with 5% PhiX.

Paired-end sequencing files for GEX and VDJ libraries were aligned to the murine reference genome (mm10) and V(D)J germlines (GRCm38) using 10x Genomics cellranger (v4.0.0) count and vdj arguments, respectively. The filtered feature matrix directory was supplied as input to the automate_GEX function in the R package Platypus (v2.0.5) (13), which uses the transcriptome analysis workflow of the R package Seurat (41). Only those cells containing less than 20% of mitochondrial reads were retained in the analysis. Genes involved in the adaptive immune receptor (e.g., TRB, TRBV1-1), were removed from the count matrix to prevent clonal relationships from influencing transcriptional phenotypes. Gene expression was normalized using the “scale.data” argument in automate_GEX, which first performs log-normalization with a scaling factor of 10000 and then scales mean expression and variance to 0 and 1, respectively. 2000 variable features were selected using the “vst” selection method and used as input to principal component analysis (PCA) using the first 10 dimensions. Graph-based clustering using the Louvain modularity optimization and hierarchical clustering was performed using the functions FindNeighbors and FindClusters in Seurat using the first ten dimensions and a cluster resolution of 0.5. UMAP was similarly inferred using the first ten dimensions. The FindMarkers function from Seurat was used when calculating differentially expressed genes (both across groups or across clusters) with both the minimum log fold change and the minimum number of cells expressing each gene set to 0.25. Mitochondrial and ribosomal genes were removed when either visualizing DE genes or supplying the top DE genes as input to gene ontology and gene set enrichment analyses. Cluster 9, which contained B cells, was removed for further analysis. Gene ontology and gene set enrichment analysis was performed using the GEX_GOterm and GEX_GSEA functions in Platypus by supplying either the top N or bottom N genes as input. In the case of GEX_GSEA, the C7 immunological signatures gene set from the Broad institute was supplied as input to the function (42). The GEX_GSEA function uses the R package fgsea (43) to conduct gene set enrichment analysis and GEX_GOterm is based on the R package edgeR (44). Projection of cells onto a reference UMAP was done using the R package ProjecTILs (25). Each infection condition was projected individually onto the acute and chronic viral infection CD8 T cell atlas and the tumor-infiltrating T lymphocytes (TIL) atlas.

For TCR repertoire analysis, the output directory of 10x Genomics cellranger vdj function was supplied as input to the VDJ_analyze function in Platypus maintaining the default clonotyping strategy (CDRa3+CDRb3 nucleotide sequence) as performed by cellranger. Those clones not containing exactly one TRA and one TRB chain were removed from the analysis. Clonal frequency was determined by counting the number of distinct cell barcodes for each unique CDR3. Overlap matrices were calculated by first appending the CDRa3 and CDRb3 nucleotide sequences and then quantifying the exact matches across repertoires. Similarity networks were calculated based on the VDJ_network function in Platypus, which first calculates the edit distance separately for TRB and TRA CDR3s, and then draws edges between those clones with a distance below the specified threshold. Circos plots were created using the VDJ_circos function in Platypus with a label.threshold of 5. Those cells in clones supported by only one cell were considered lowly expanded clones, whereas those clones supported by two or more cells were considered expanded. Logo plots were created using the VDJ_logoplot_vector function in Platypus on unique CDRa3 and CDRb3 sequences.

### Data Visualization

Heatmaps displaying differential gene expression were produced using the DoHeatmap function in the R package Seurat (v4.0.1) (45). Gene enrichment analysis was performed using the GEX_GOterm function in Platypus, which is based on the analysis pipeline in edgeR (44). Enrichment plots were produced using the R package ggplot (46). Gene set enrichment analysis was performed using the GEX_GSEA function in Platypus (v3.1) under default parameters, which utilizes fgsea (v1.12), tibble (v2.1.3), and the C7 gene set from the molecular signatures database MSigDB (43, 47). Similarity networks were produced using the R package igraph (48). Circos plots were produced using the chordDiagram function of the R package Circlize (50). Logoplots were produced using the R packages ggseqlogo (49). All other figures were produced using Prism v9 (Graphpad).

## Data Availability Statement

The datasets presented in this study can be found in online repositories. The names of the repository/repositories and accession number(s) can be found below: European Bioinformatics Institute, E-MTAB-11330.

## Ethics Statement

The animal study was reviewed and approved by veterinary office of the canton of Zurich under animal experimentation licenses 115/2017 and ZH058/20.

## Author Contributions

RK, IS, AA, K-LH, DS, DN, and AY performed experiments and analyzed data. All authors contributed to designing the study and writing the manuscript. All authors contributed to the article and approved the submitted version.

## Funding

This work was supported by the European Research Council Starting Grant 679403 (to SR), ETH Zurich Research Grants (to SR and AO), and an ETH Career Seed Grant (AY).

## Conflict of Interest

The authors declare that the research was conducted in the absence of any commercial or financial relationships that could be construed as a potential conflict of interest.

## Publisher’s Note

All claims expressed in this article are solely those of the authors and do not necessarily represent those of their affiliated organizations, or those of the publisher, the editors and the reviewers. Any product that may be evaluated in this article, or claim that may be made by its manufacturer, is not guaranteed or endorsed by the publisher.
