# Supplementary material for: Clonally Expanded Virus-Specific CD8 T Cells Acquire Diverse Transcriptional Phenotypes During Acute, Chronic, and Latent Infections
Source: Front Immunol. 2022 Feb 2;13:782441. doi: 10.3389/fimmu.2022.782441 (PMC8847396; doi:10.3389/fimmu.2022.782441)
Supplement: Supplementary file 1 [file DataSheet_1.pdf]

Supplementary Material

1 Supplementary Figures and Tables

1.1 Supplementary Figures

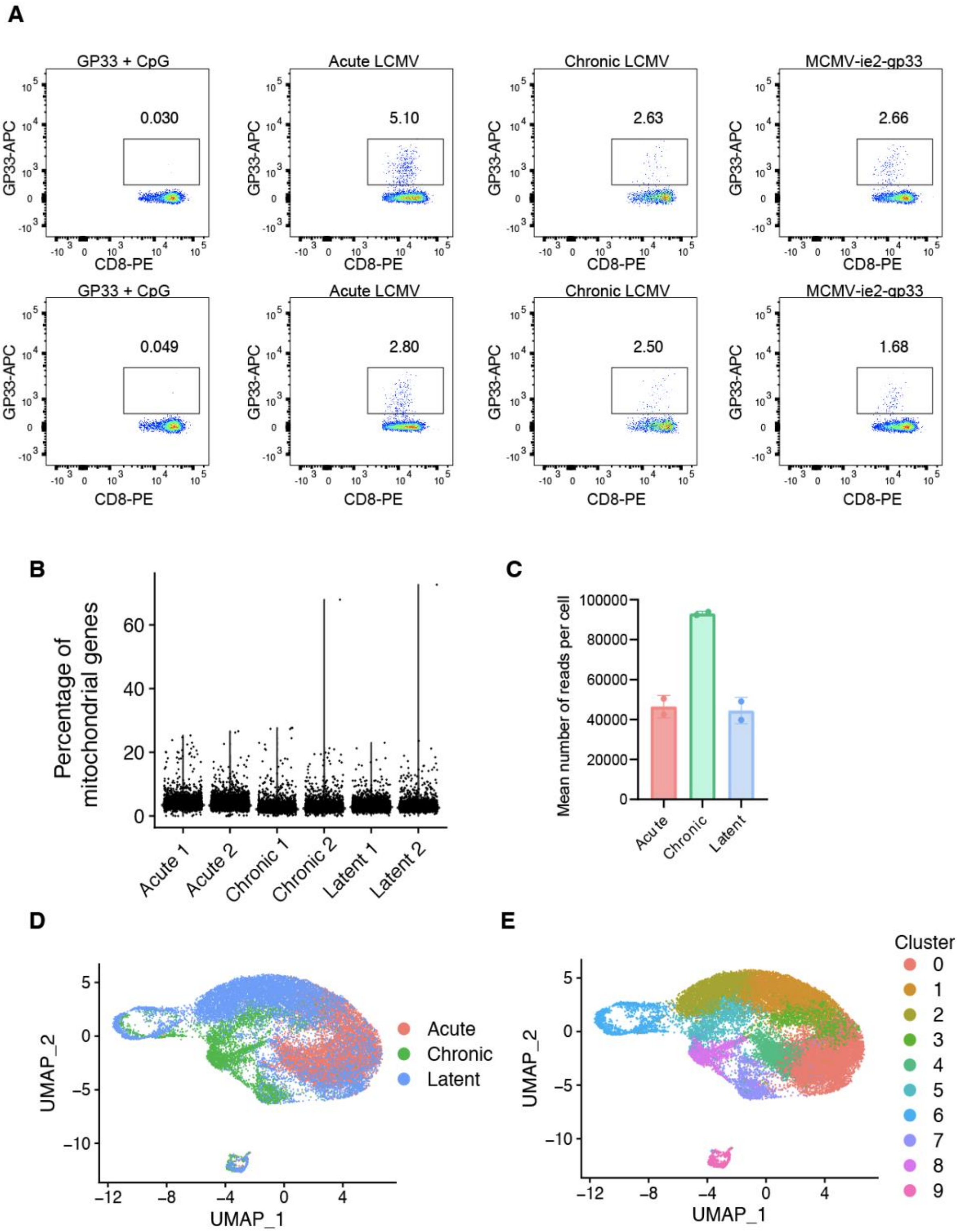

Supplementary Figure S1. Single-cell sequencing of virus-specific CD8+ T cells. A. Flow cytometry plots of tetramer-sorted GP33-specific cells for all 6 mice. Pre-gated on lymphocytes, singlets, alive+ CD8+ cells. Due to the low number of cells, single-cell sequencing of the GP33 + CpG group was not performed. B. Percentage of mitochondrial genes per cell. C. Mean number of reads per cell. D. Uniform manifold approximation projection (UMAP) colored by infection group. Each point represents a cell and color corresponds to transcriptional clusters. All cells from all samples were integrated in this single UMAP. E. UMAP colored by infection group transcriptional cluster.

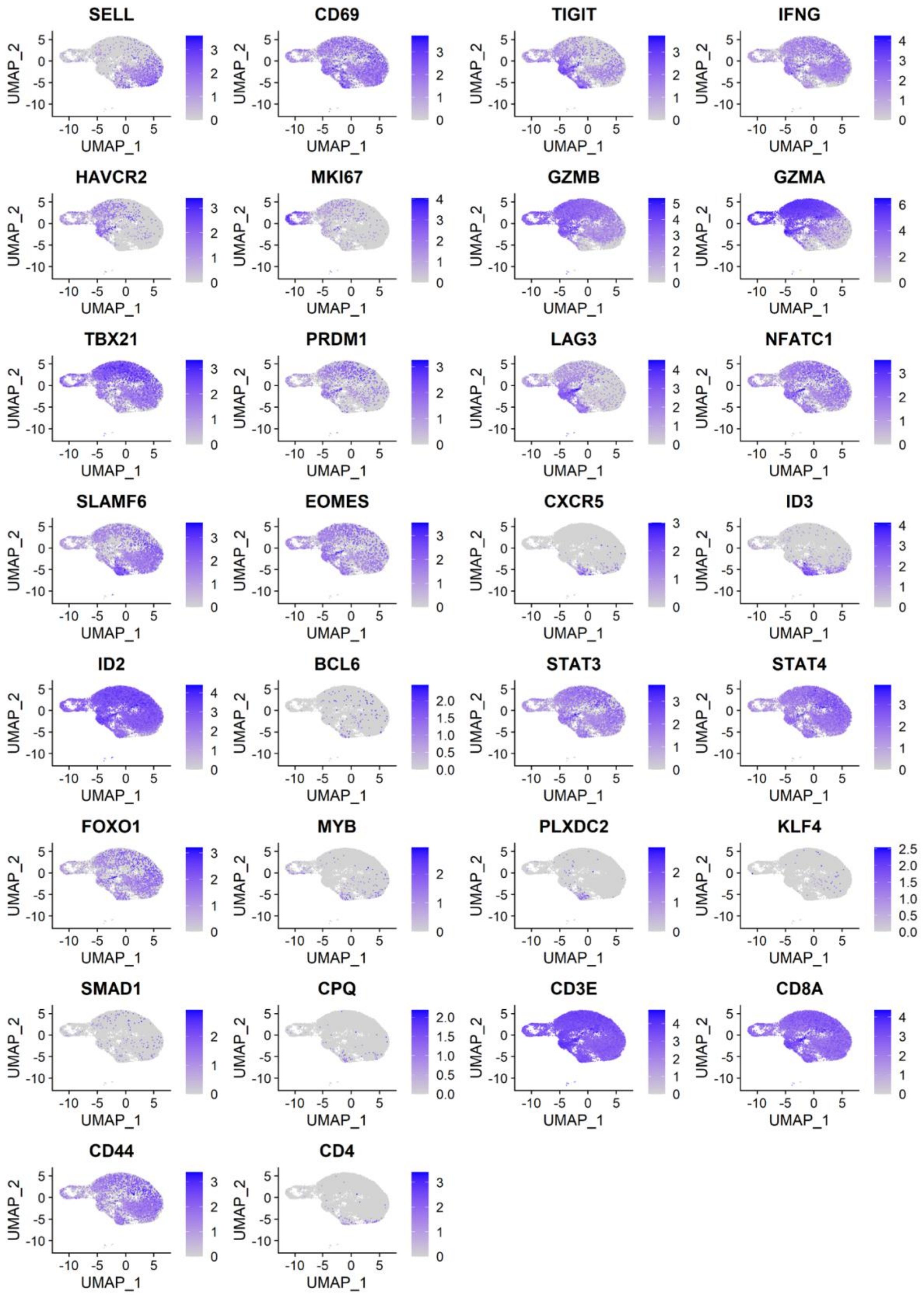

Supplementary Figure S2. Normalized gene expression for select genes of interest. All cells from all samples were integrated into a single uniform manifold approximation projection (UMAP).

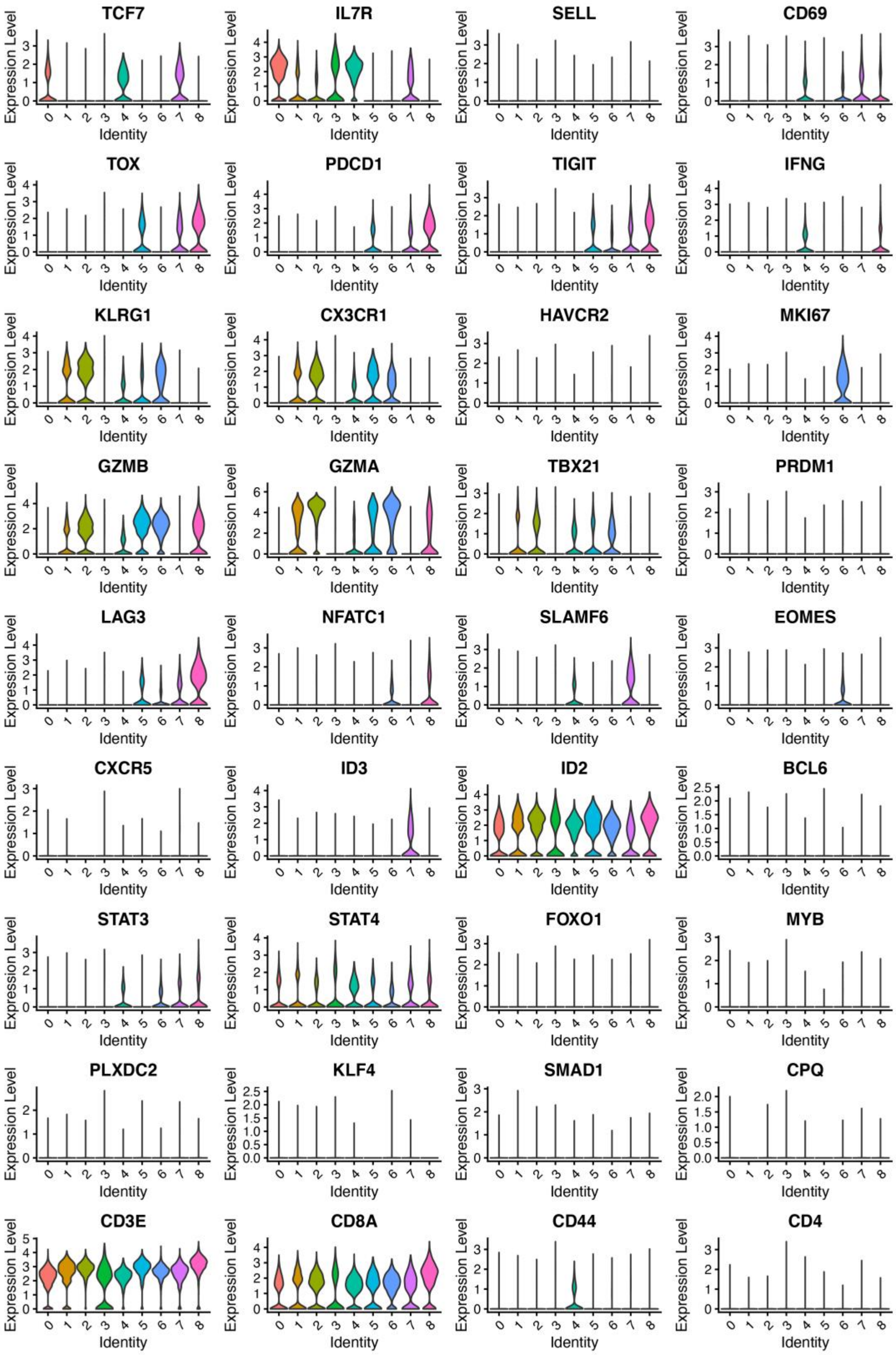

Supplementary Figure S3. Normalized gene expression for select genes of interest split by transcriptional cluster.

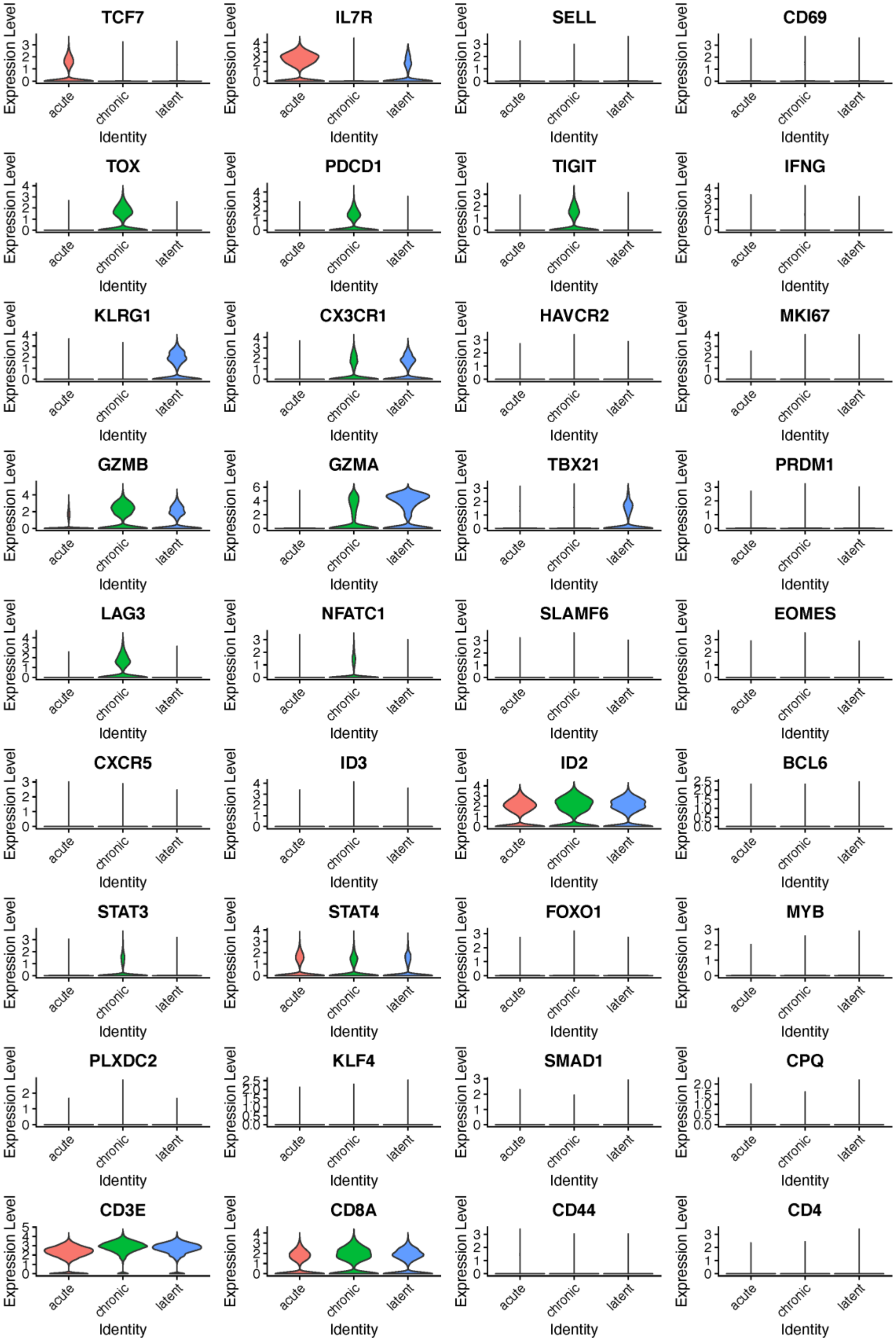

Supplementary Figure S4. Normalized gene expression for select genes of interest split by infection type.

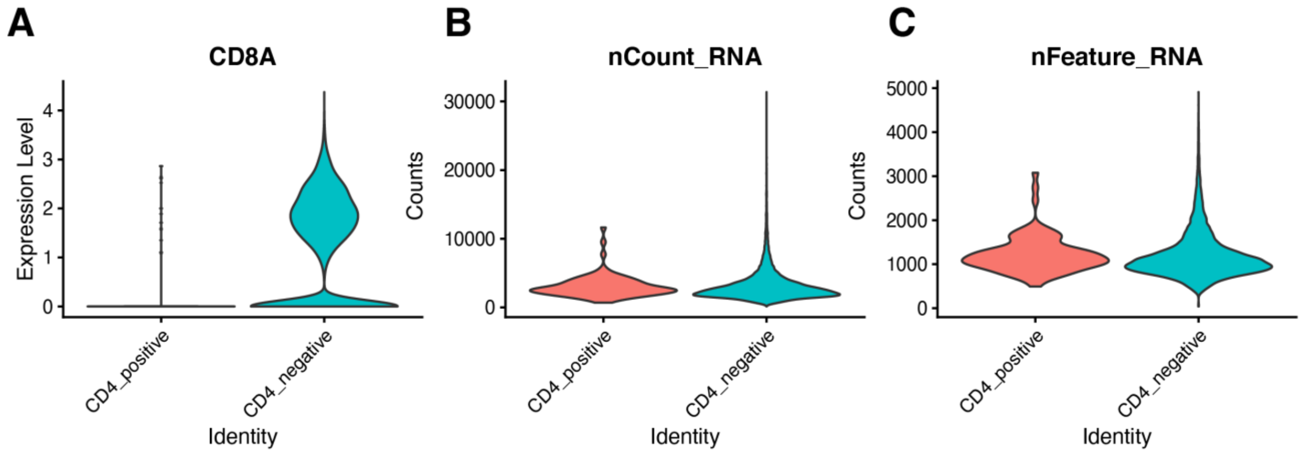

Supplementary Figure S5: Violin plots showing features of CD4 positive and negative cells. A. Normalized CD8A gene expression in CD4 positive and negative cells. B. Number of RNA molecules detected in each cell for CD4 positive and CD4 negative cells. C. Number of genes detected in each cell for CD4 positive and CD4 negative cells.

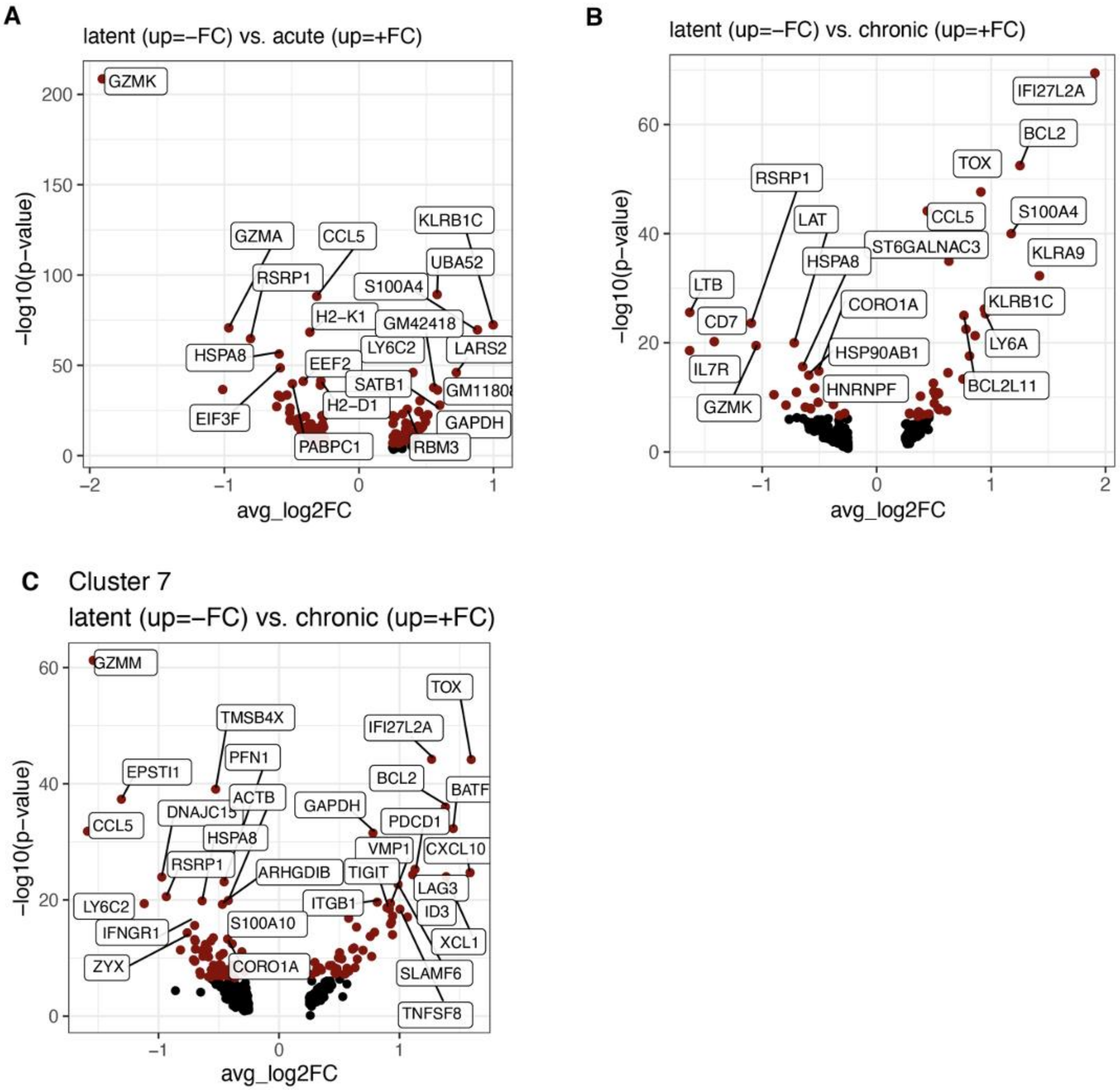

Supplementary Figure S6. Differential gene expression analysis between cells from different infection conditions within the same cluster. A. Differential gene expression between cells following acute LCMV infection and MCMV-*ie2*-gp33 infection in cluster 1. B. Differential gene expression between cells following chronic LCMV infection and MCMV-*ie2*-gp33 infection in cluster 1. C. Differential gene expression between cells following chronic LCMV infection and MCMV-*ie2*-gp33 infection in cluster 7. Points in red indicate significantly differentially expressed genes.

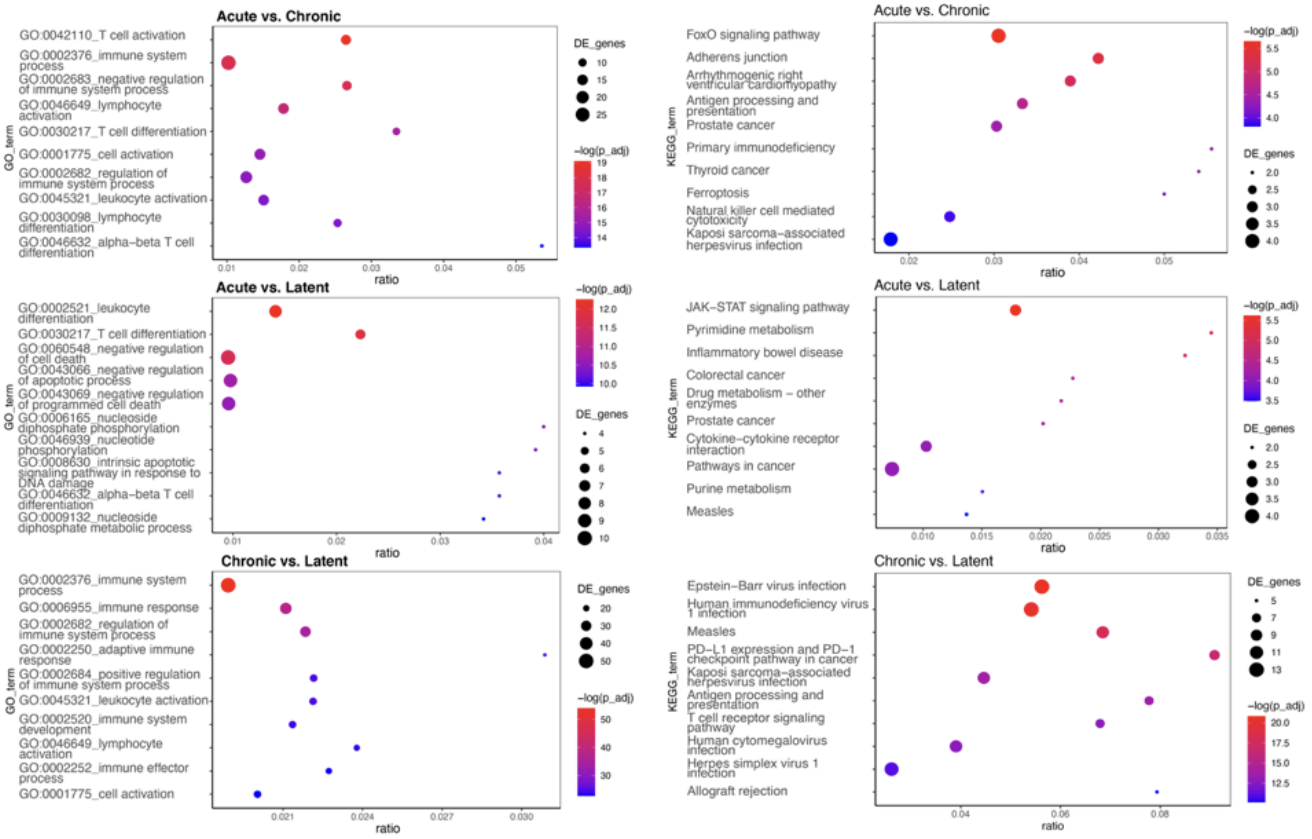

Supplementary Figure S7. Gene ontology (GO) term (left panel) enrichment and pathway analysis (right panel) of the 10 most upregulated genes from either acute versus chronic LCMV infection (top), acute LCMV versus MCMV-*ie2*-gp33 infection (middle), or chronic versus MCMV-*ie2*-gp33 (bottom) infection. The color of each dot corresponds to adjusted p value. The size of the dot corresponds to the number of genes. Ratio corresponds to the number of differentially genes relative to the number of total genes corresponding to each GO term.

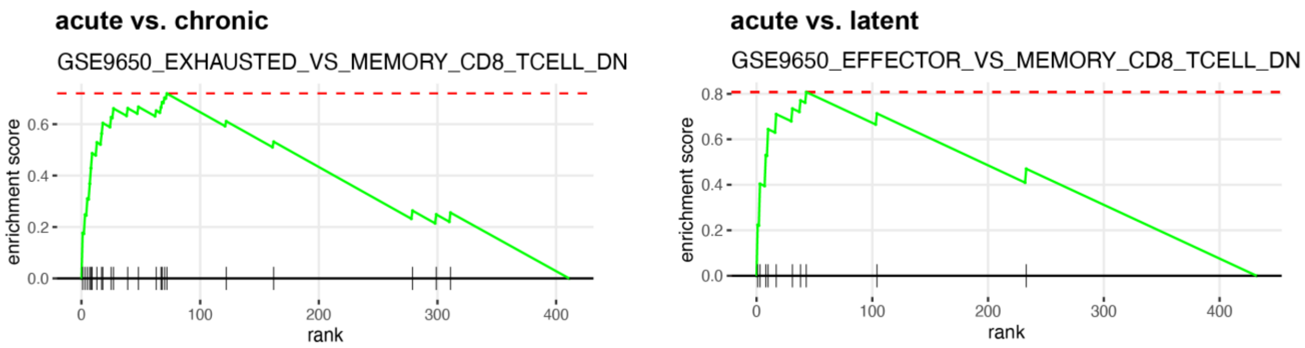

Supplementary Figure S8. Gene set enrichment (GSEA) plots based on the C7 immunological signatures from the Broad institute. The upregulated genes from either acute versus chronic LCMV infection or acute versus MCMV-*ie2*-gp33 infection were supplied as input.

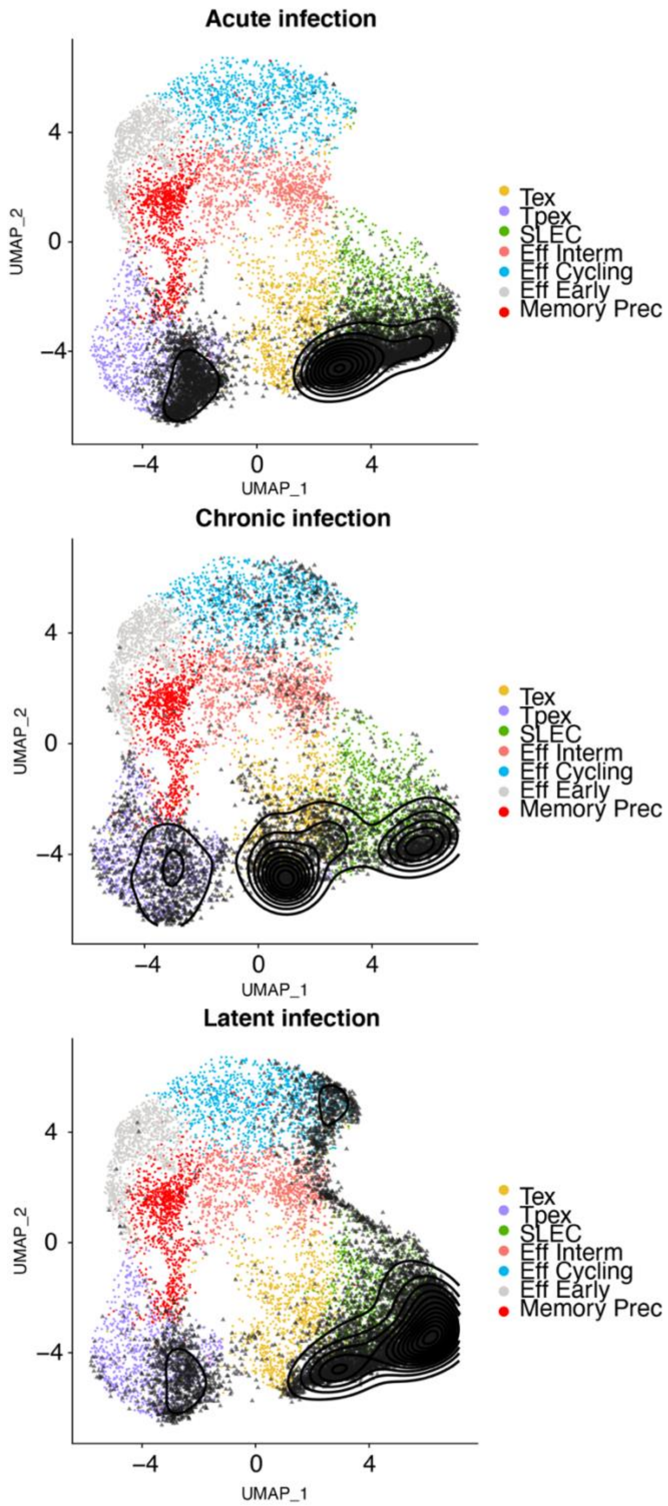

Supplementary Figure S9: Uniform manifold approximation projection (UMAP) showing cells projected onto the acute and chronic viral infection CD8 T cell atlas reference from ProjecTILs with annotated functional clusters. Cells from acute LCMV (top), chronic LCMV (middle) and MCMV-ie2-gp33 (bottom) were projected separately.

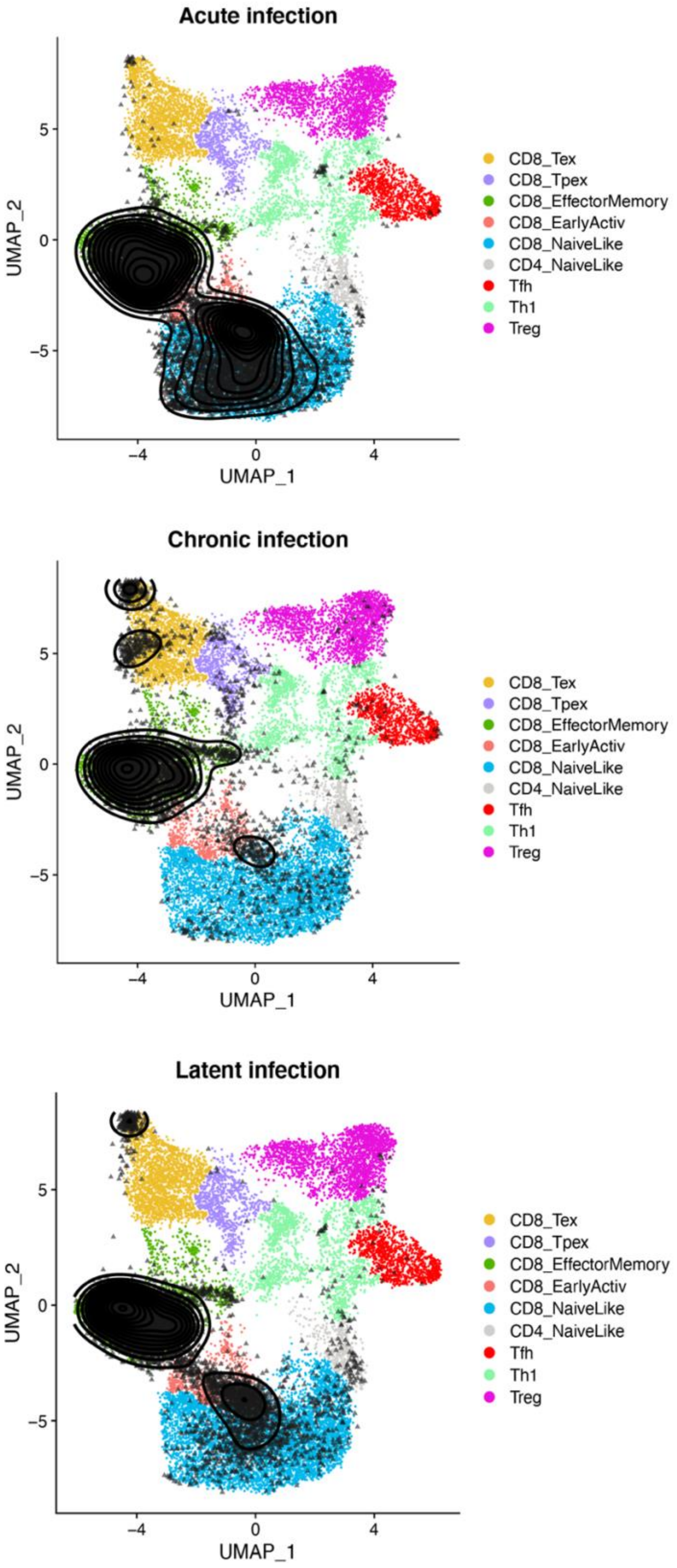

Supplementary Figure S10: Uniform manifold approximation projection (UMAP) showing cells projected onto the tumor-infiltrating T lymphocytes (TIL) atlas reference from ProjecTILs with annotated functional clusters. Cells from acute LCMV (top), chronic LCMV (middle) and MCMV-ie2-gp33 (bottom) were projected separately.

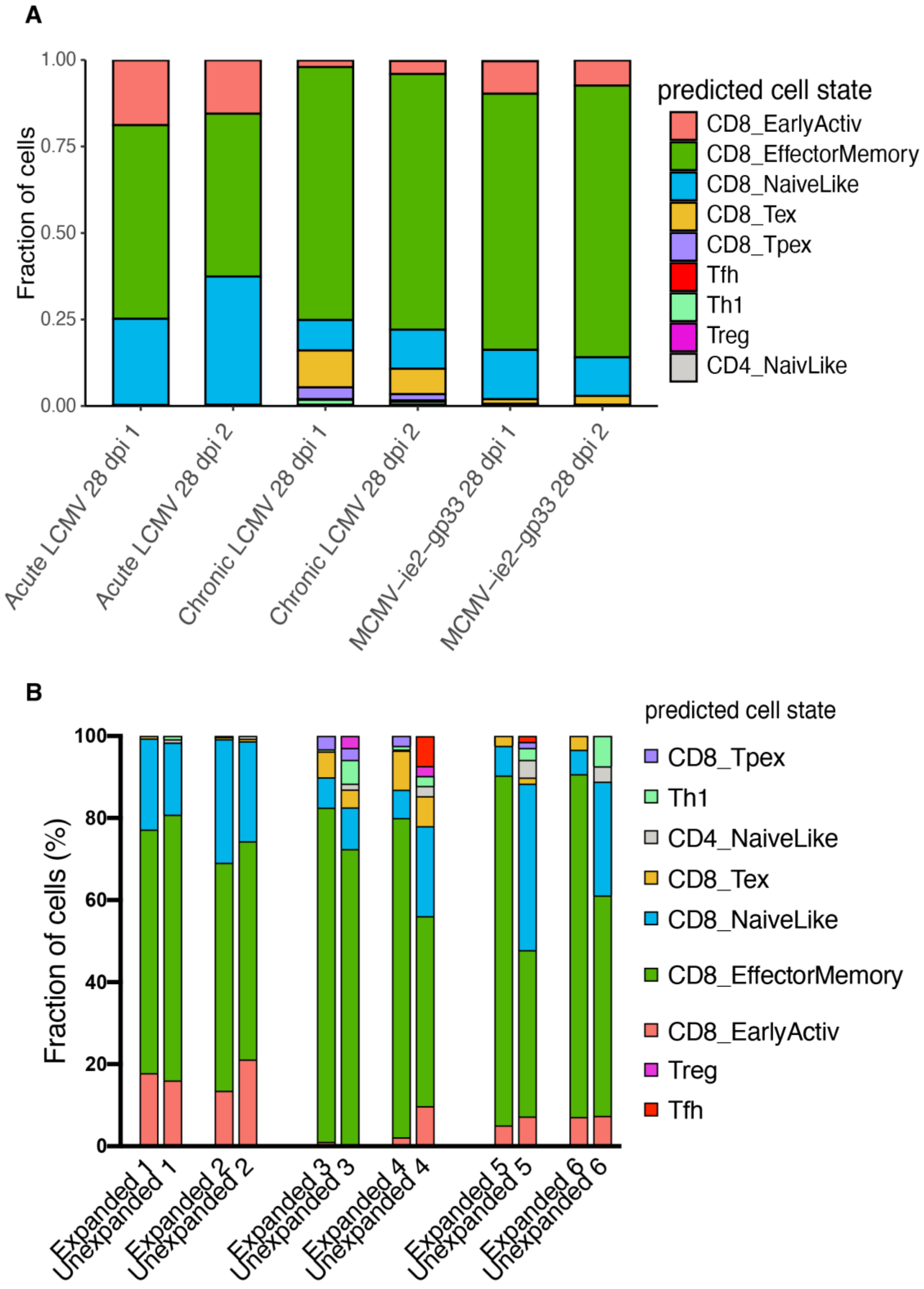

Supplementary Figure S11: A. Fraction of cells belonging to functional clusters determined by ProjecTILs projected on the tumor-infiltrating T lymphocytes (TIL) atlas. B. Functional cluster membership determined by ProjectTILs using the tumor-infiltrating T lymphocytes (TIL) atlas for expanded (+) and lowly expanded (-) clones for each mouse. Lowly expanded clones were those clones supported by only one unique cell barcode.

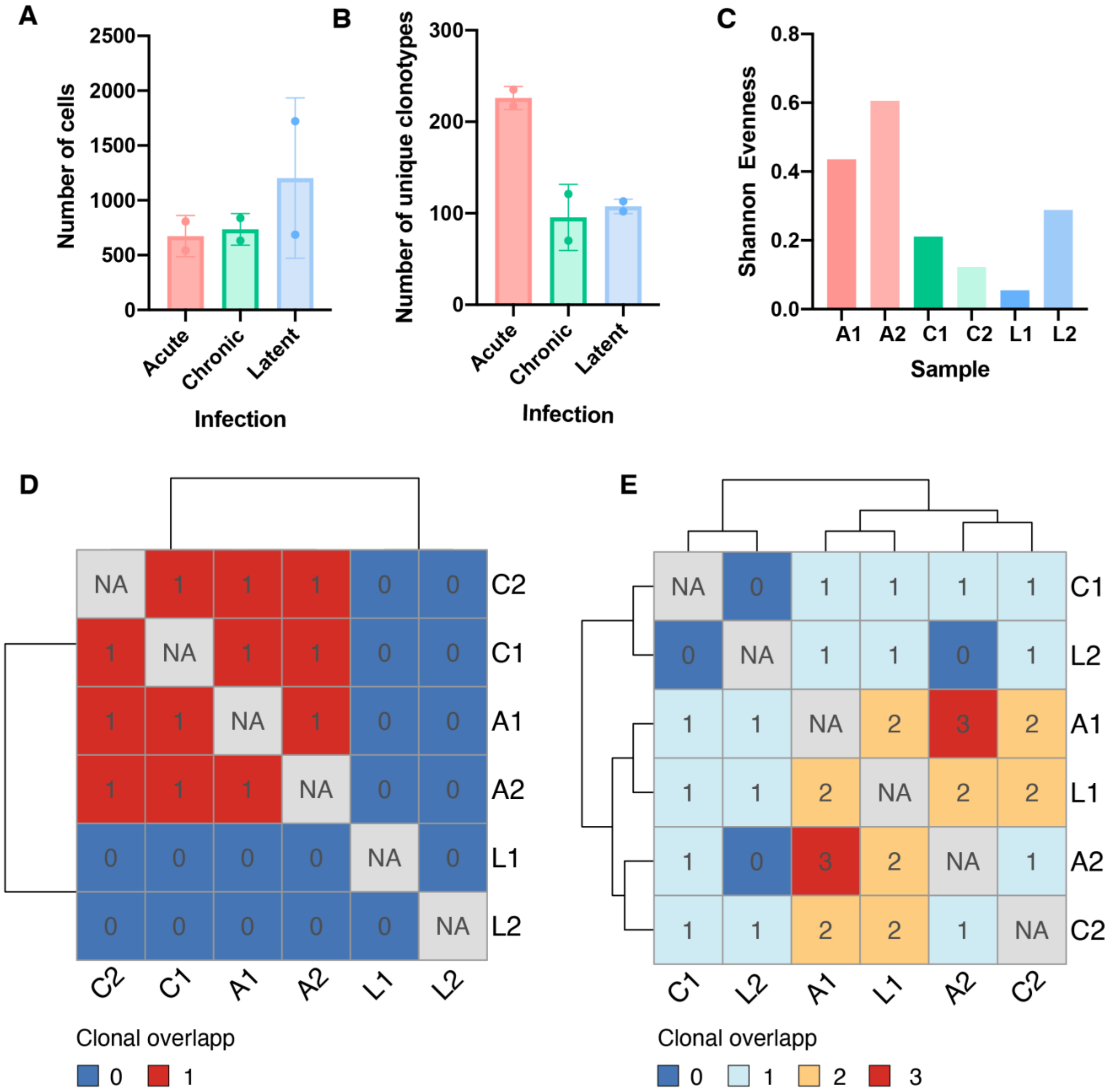

Supplementary Figure S12. T cell receptor repertoire features of virus-specific CD8+ T cells following acute, chronic, and latent viral infection. A. Number of recovered GP33-specific cells containing exactly one T cell receptor beta (TRB) and T cell receptor alpha (TRA) B. Number of unique clones per repertoire. Clone was defined by identical CDRb3-CDRa3 nucleotide sequence. C. Shannon evenness quantifying the distribution of clonal frequency for mice infected with either acute (A1, A2), chronic (C1, C2), or latent (L1, L2) infection. D. Heatmap showing pairwise clonal overlap for the top 10 most expanded GP33-specific T cell clones from each mouse. E. Heatmap showing pairwise clonal overlap for those clones supported by two or more distinct cell barcodes.

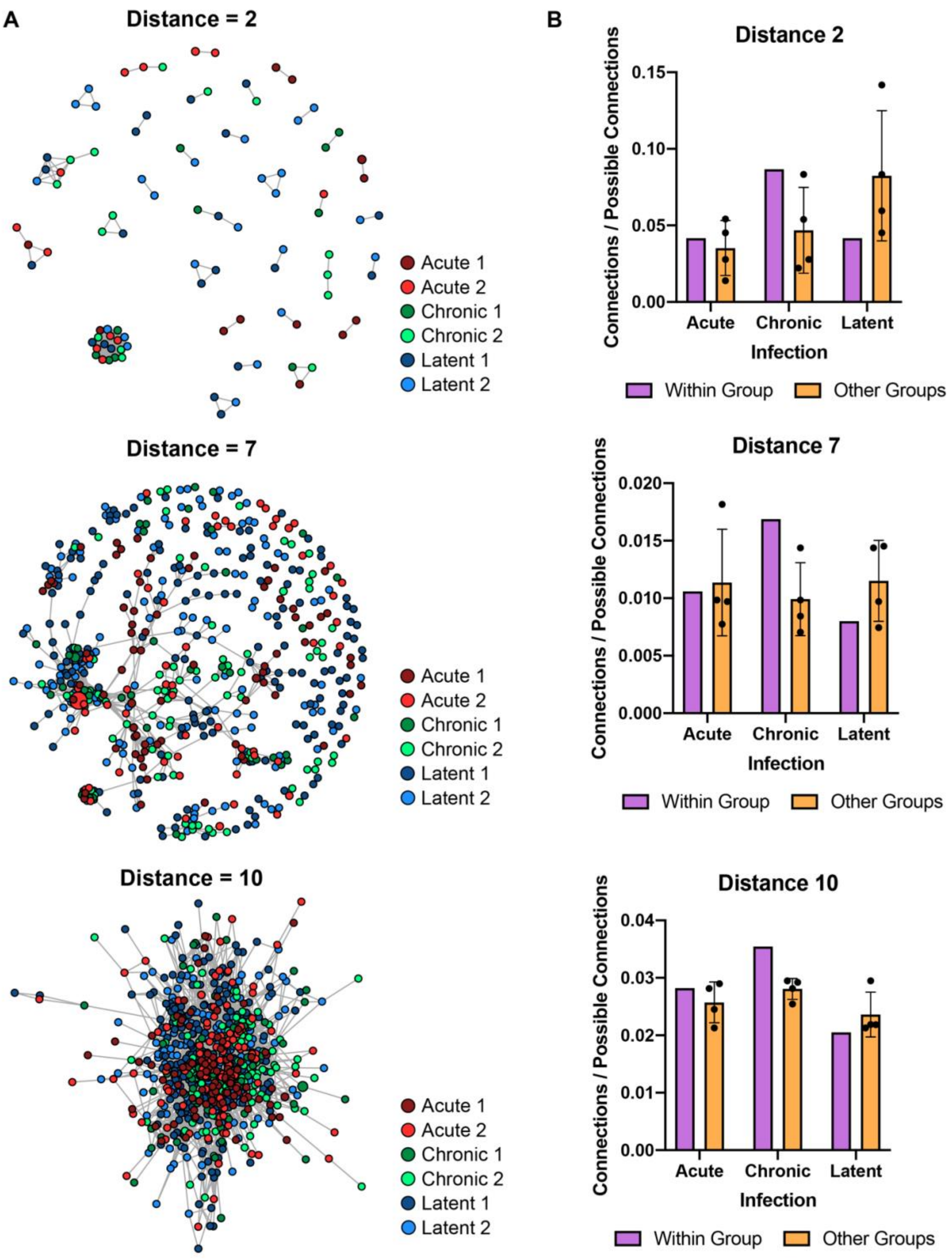

Supplementary Figure S13. Similarity networks with diverse amino acid edit distance thresholds. A. Similarity network of virus-specific CD8+ T cell clones. Nodes represent a unique CDRb3-CDRa3 from each mouse. Edges connect those clones separated by an edit distance of N amino acids or less. B. The number of edges between nodes of mice either in the same infection group or with different infection groups normalized by the number of possible connections.

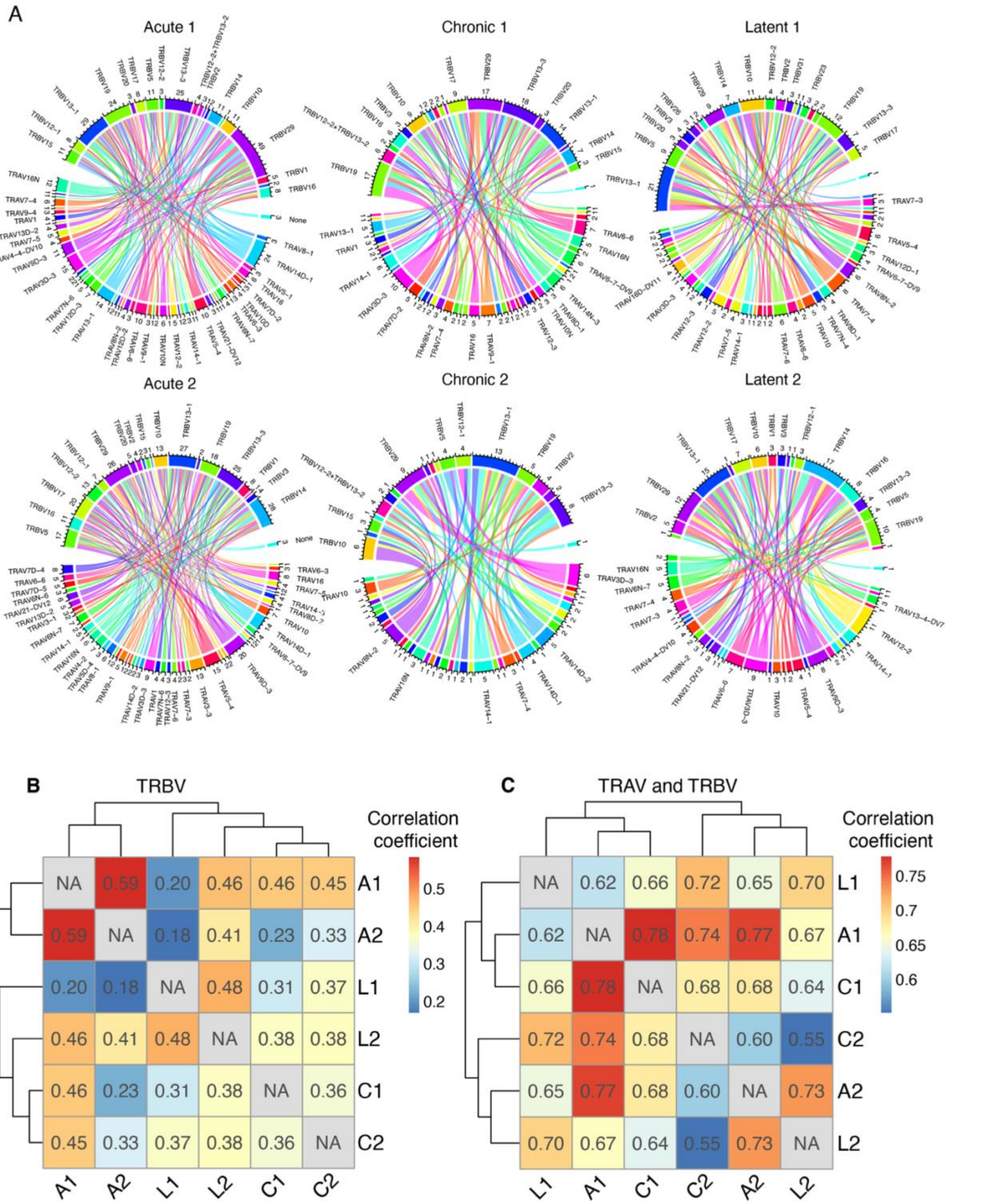

Supplementary Figure S14. Germline gene usage of GP33-specific CD8+ T cell receptors. A. Circos plots depicting the relationship between TRB and TRA V gene usage. Color corresponds to TRA gene usage. Connections illustrate the number of clones using each particular combination. Clone was defined as an identical CDRb3-CDRa3 nucleotide sequence. B. Correlation heatmap quantifying the fraction of unique clones using a particular TRB V gene. Intensity corresponds to the Pearson correlation of the V gene usage vector between two samples. C. Correlation heatmap quantifying the fraction of unique clones using a particular TRA and TRB V gene combination. Intensity corresponds to the Pearson correlation of the V gene usage vector between two samples.

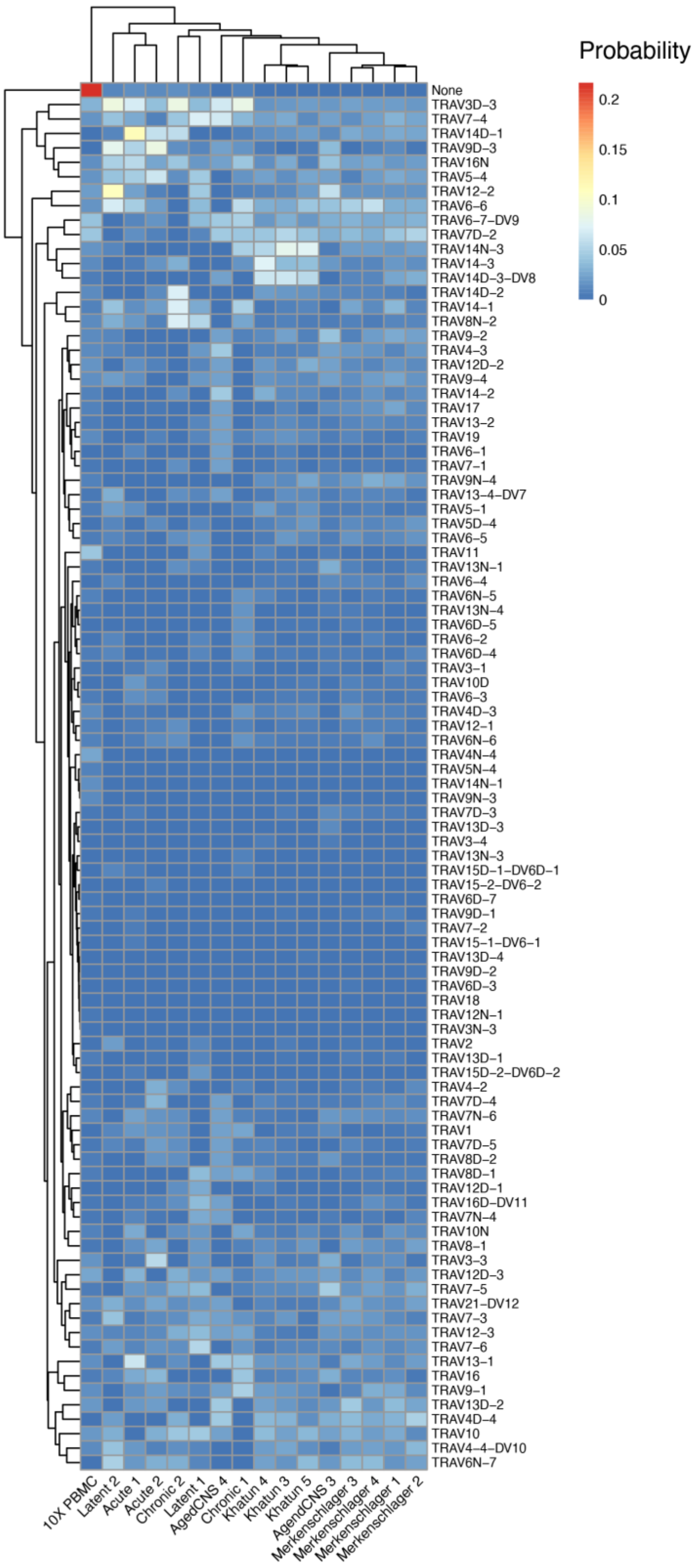

Supplementary Figure S15: TRA V gene usage compared to other single-cell immune repertoire sequencing datasets containing naive and CD4 T cells.

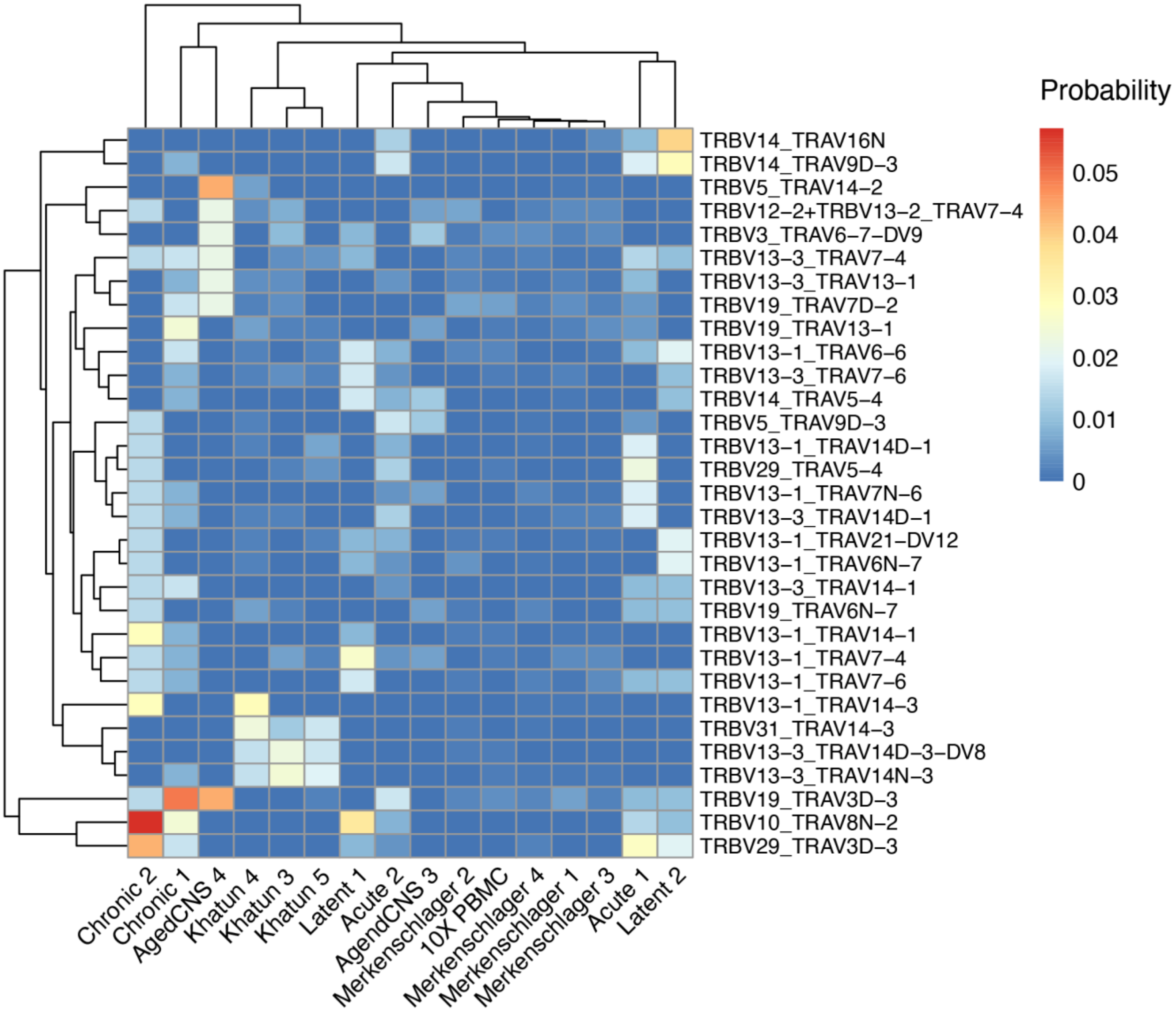

Supplementary Figure S16: Pasted TRA_TRB TRA V gene usage compared to other single-cell immune repertoire sequencing datasets containing naive and CD4 T cells. Only gene combinations with a combined probability larger 0.005 are shown.

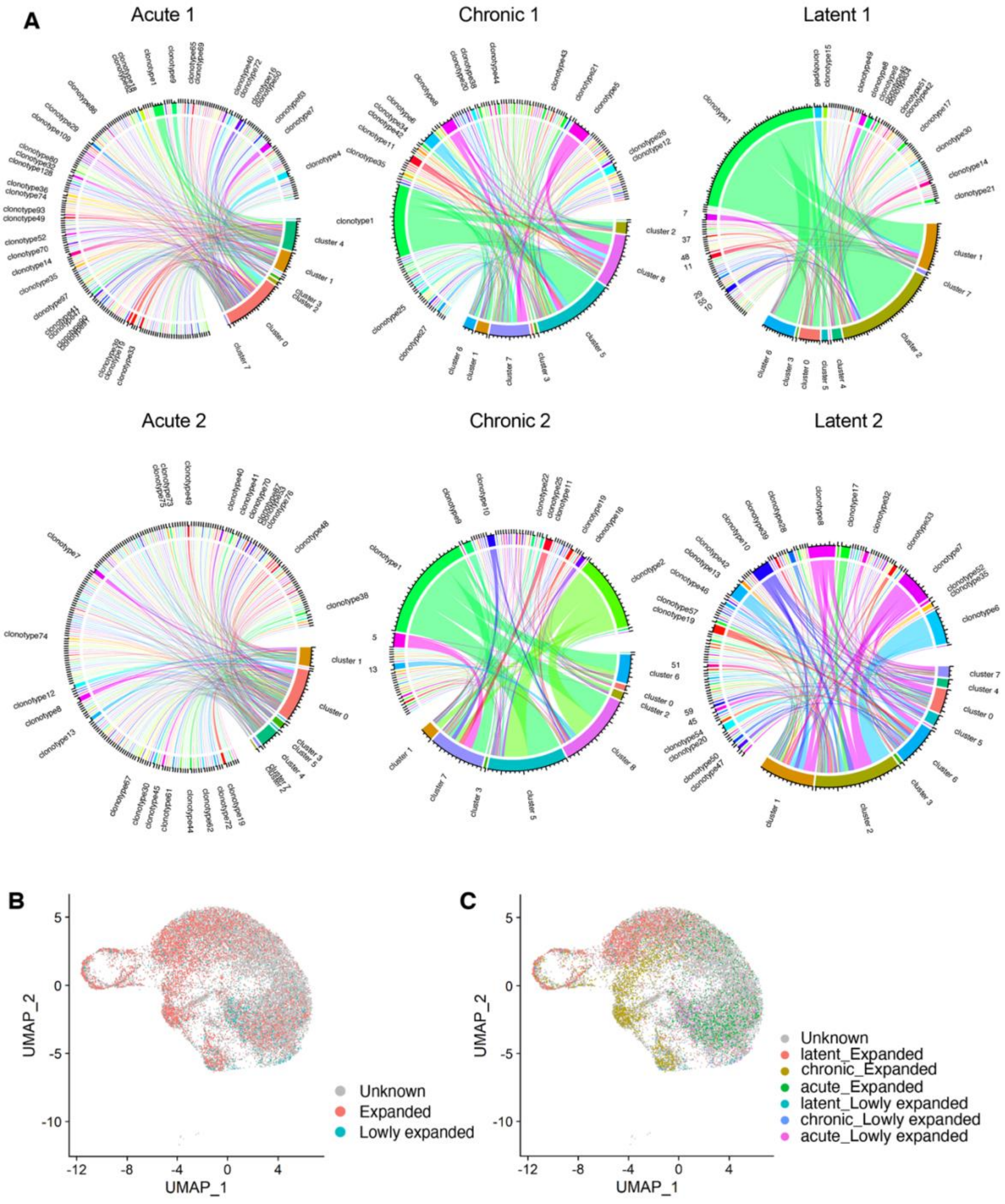

Supplementary Figure S17. Supporting information for cluster membership. A. Circos plots relating cluster membership to clonal expansion. Color corresponds to each unique clone. Connections illustrate the number of clones using each particular combination. Clone was defined as an identical CDRb3-CDRa3 nucleotide sequence. C. Uniform manifold approximation projection (UMAP) showing expanded (>1 cell) and lowly expanded (1 cell) clones. Each point is a cell and cells from all infection conditions and mice were pooled. D. UMAP visualizing expanded and lowly expanded clones but colored by infection type.

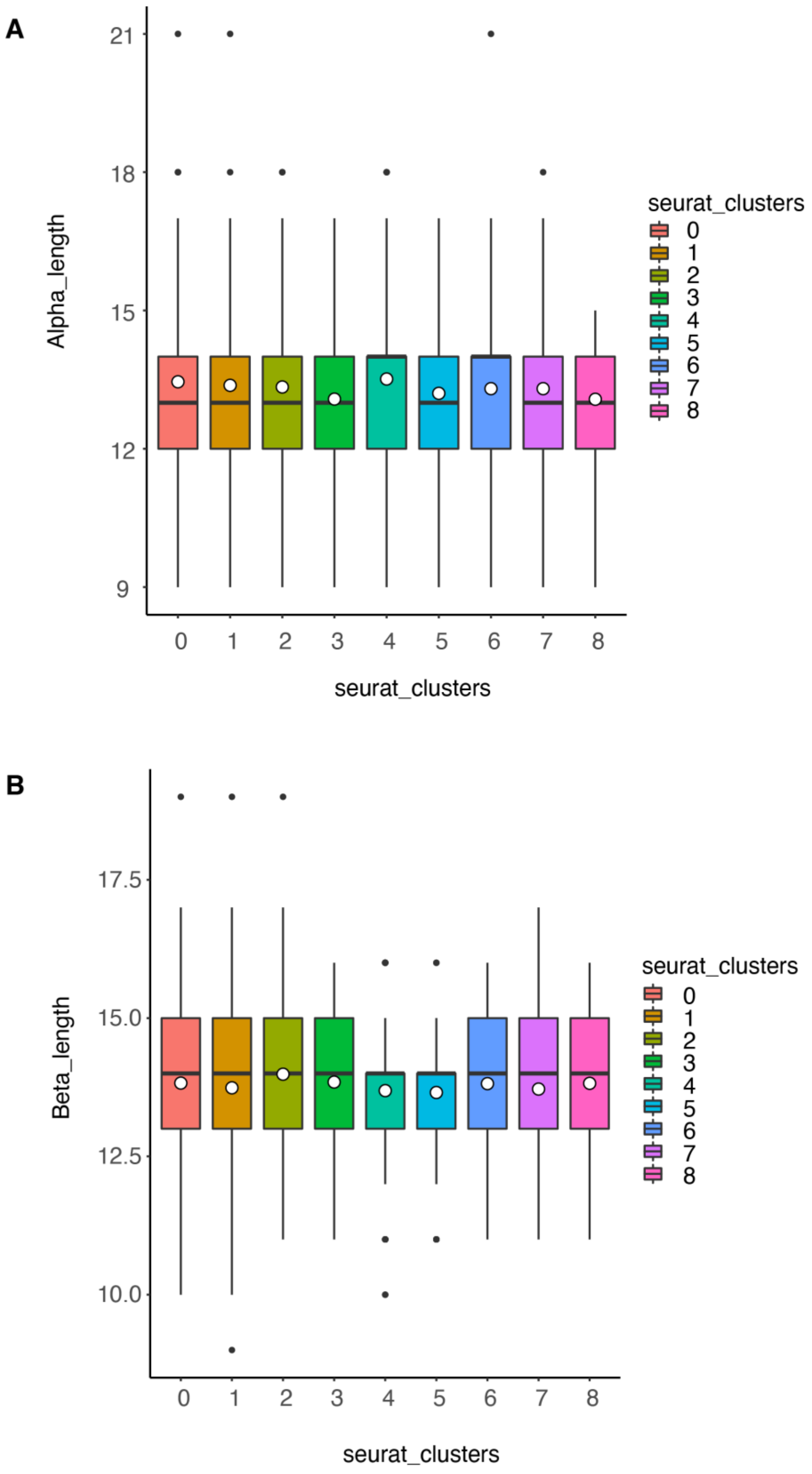

Supplementary Figure S18. CDR3 length distribution of unique clonotypes across transcrprional clusters. A. CDR3 length of the TCR alpha chain. B. CDR3 length of the TCR beta chain. Black bar shows median CDR3 length, white circle shows mean CDR3 length.

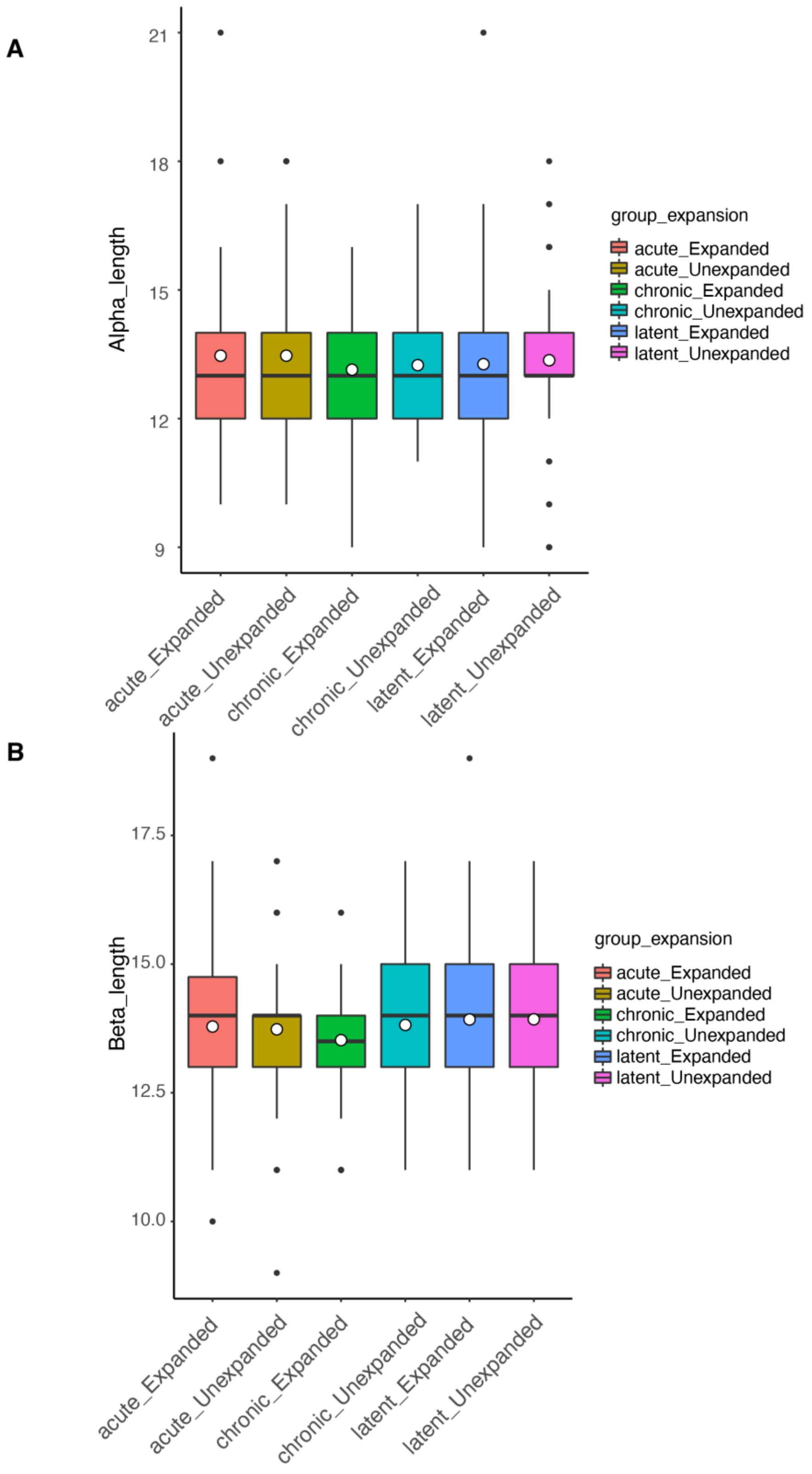

Supplementary Figure S19. CDR3 length distribution of unique expanded and lowly expanded clonotypes across infection conditions. A. CDR3 length of the TCR alpha chain. B. CDR3 length of the TCR beta chain. Black bar shows median CDR3 length, white circle shows mean CDR3 length.

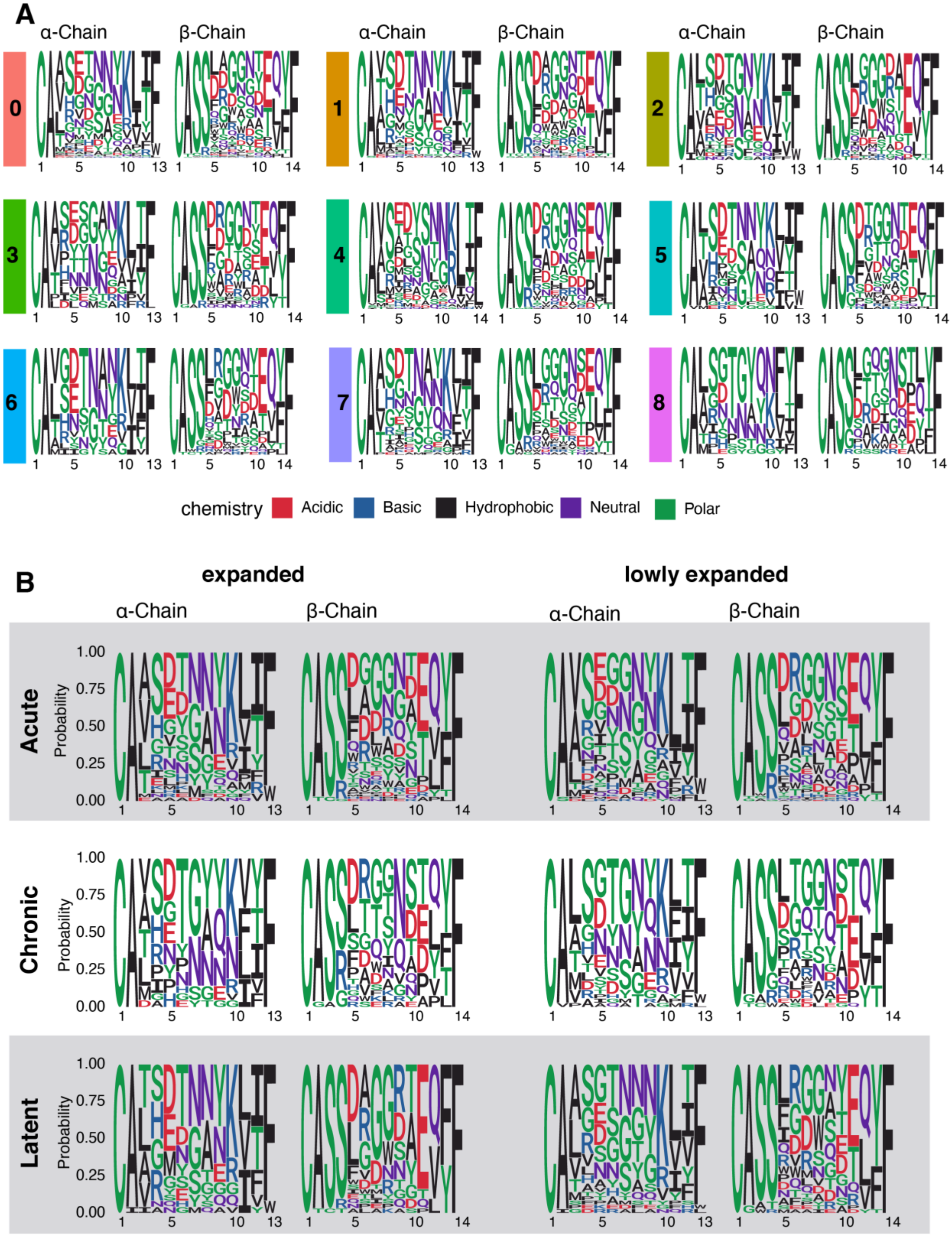

Supplementary Figure S20. CDR3 motifs of TCR Alpha and Beta chains show residue enrichment in expanded clones. A. Logoplots of CDR3s per transcriptional cluster. B. Logoplots of CDR3s per infection type for expanded and lowly expanded clones. CDR3s of unique clonotypes are plotted. Mean CDR3 length as determined in Supplementary Figure S18 and S19 was plotted for each cluster and chain.

1.2 Supplementary Tables

	P_VAL	AVG_LOGFC	PCT.1	PCT.2	P_VAL_ADJ
**IL7R**	0	2.5820406	0.763	0.117	0
**DNAJC15**	0	1.14657743	0.765	0.462	0
**TAGLN2**	0	1.08326259	0.765	0.479	0
**LY6C2**	0	1.06412692	0.962	0.591	0
**NME2**	0	0.74840178	0.876	0.737	0
**TMSB10**	0	0.50303587	0.998	0.988	0
**TPT1**	0	0.44951551	0.995	0.978	0
**H2-K1**	0	-0.5165216	0.985	0.993	0
**CASP3**	0	-0.7813788	0.049	0.273	0
**CD3E**	0	-0.8404149	0.884	0.963	0
**IKZF3**	0	-0.9616957	0.227	0.553	0
**RTP4**	0	-1.0676769	0.062	0.341	0
**VMP1**	0	-1.0974053	0.191	0.526	0
**NKG7**	0	-1.1020099	0.958	0.966	0
**AW112010**	0	-1.1190745	0.92	0.961	0
**STAT1**	0	-1.1725178	0.197	0.513	0
**LGALS3**	0	-1.2694752	0.168	0.45	0
**SERPINA3G**	0	-1.3178316	0.029	0.329	0
**TIGIT**	0	-1.3419467	0.072	0.425	0
**ISG15**	0	-1.354208	0.088	0.358	0
**GZMK**	0	-1.5736839	0.191	0.543	0
**CCL3**	0	-1.7002707	0.054	0.323	0
**PDCD1**	0	-1.7954265	0.022	0.47	0
**GZMB**	0	-1.8887764	0.252	0.621	0
**TOX**	0	-2.0530245	0.02	0.561	0
**LAG3**	0	-2.1148334	0.011	0.502	0
**IFI27L2A**	0	-3.815983	0.225	0.902	0
**H2-T22**	3.74E-290	-0.8770408	0.44	0.72	1.18E-285
**UBA52**	2.24E-286	0.4889524	0.966	0.942	7.08E-282
**CD8B1**	2.83E-283	0.59085643	0.948	0.853	8.94E-279
**CD3G**	8.35E-271	-0.7408818	0.847	0.928	2.64E-266
**RGS1**	3.23E-263	-1.0899586	0.088	0.313	1.02E-258
**PSMB8**	3.57E-260	-0.6371833	0.789	0.901	1.13E-255
**UCP2**	1.82E-255	-0.7384948	0.684	0.848	5.74E-251
**SH2D2A**	8.80E-241	-0.9927662	0.3	0.56	2.78E-236
**H2-Q4**	1.93E-236	-0.8087924	0.378	0.655	6.11E-232
**INPP4B**	6.22E-234	-0.8205868	0.084	0.294	1.97E-229
**ARL6IP1**	6.35E-233	-0.874718	0.52	0.741	2.01E-228
**BATF**	9.89E-233	-0.9388381	0.187	0.441	3.13E-228
**RACK1**	2.80E-225	0.54833763	0.913	0.869	8.86E-221
**GZMA**	2.03E-220	-1.855199	0.189	0.422	6.44E-216
**STARD3NL**	3.42E-218	-0.655253	0.093	0.303	1.08E-213
**ADAM19**	9.62E-216	-0.8584745	0.1	0.308	3.04E-211
**XAF1**	1.02E-215	-0.7093301	0.086	0.29	3.22E-211
**GIMAP7**	4.86E-211	-0.8138269	0.405	0.642	1.54E-206
**ANXA2**	5.79E-209	-0.9409372	0.261	0.5	1.83E-204
**ARL4C**	4.70E-207	0.95197094	0.432	0.18	1.49E-202
**H2-D1**	1.34E-202	-0.3872465	0.98	0.992	4.24E-198
**FASL**	6.99E-202	-0.8278187	0.123	0.334	2.21E-197

Supplementary Table S1: Differentially expressed genes between cells from acute LCMV and chronic LCMV. 50 most significant genes are shown.

	P_VAL	AVG_LOGFC	PCT.1	PCT.2	P_VAL_ADJ
**IL7R**	0	1.2715417	0.763	0.37	0
**LARS2**	0	1.05214035	0.878	0.714	0
**BCL2**	0	1.04193781	0.385	0.106	0
**UBA52**	0	0.86666836	0.966	0.903	0
**IER3**	0	0.85055062	0.286	0.075	0
**GM11808**	0	0.84528316	0.809	0.641	0
**TPT1**	0	0.36099932	0.995	0.993	0
**H2-K1**	0	-0.4350025	0.985	0.995	0
**H2-D1**	0	-0.5136601	0.98	0.994	0
**RAC2**	0	-0.5279979	0.946	0.981	0
**CCL5**	0	-0.5381424	0.994	0.985	0
**PFN1**	0	-0.5499805	0.987	0.995	0
**ARPC2**	0	-0.5840303	0.78	0.885	0
**CD3E**	0	-0.5892626	0.884	0.956	0
**EMP3**	0	-0.6251103	0.769	0.895	0
**HSPA8**	0	-0.6288198	0.832	0.916	0
**ACTB**	0	-0.6517192	0.997	0.999	0
**ITM2B**	0	-0.665355	0.849	0.944	0
**CTSD**	0	-0.7073449	0.646	0.821	0
**YWHAZ**	0	-0.7105418	0.639	0.822	0
**ACTG1**	0	-0.7295447	0.926	0.964	0
**ITGB2**	0	-0.7576575	0.511	0.731	0
**NKG7**	0	-0.7896016	0.958	0.978	0
**CD48**	0	-0.8957331	0.55	0.777	0
**ANXA2**	0	-0.9379661	0.261	0.494	0
**ZEB2**	0	-0.9736992	0.202	0.518	0
**LGALS1**	0	-0.9849358	0.885	0.925	0
**PYCARD**	0	-1.0223589	0.368	0.614	0
**H2AFZ**	0	-1.0422756	0.796	0.919	0
**REEP5**	0	-1.0624103	0.412	0.69	0
**VIM**	0	-1.0693859	0.649	0.788	0
**CX3CR1**	0	-1.0716379	0.193	0.506	0
**S1PR5**	0	-1.1426206	0.14	0.448	0
**KLRG1**	0	-1.2141242	0.224	0.579	0
**STMN1**	0	-1.3457496	0.072	0.288	0
**GZMB**	0	-1.3634889	0.252	0.548	0
**LGALS3**	0	-1.3792493	0.168	0.485	0
**GZMK**	0	-2.2273265	0.191	0.702	0
**GZMA**	0	-3.0198733	0.189	0.713	0
**CDC42**	7.62E-306	-0.5801699	0.705	0.837	2.41E-301
**LCK**	1.74E-305	-0.4899978	0.838	0.929	5.51E-301
**LAPTM5**	2.57E-303	-0.4658568	0.905	0.955	8.12E-299
**ACTR3**	8.12E-303	-0.6253719	0.649	0.794	2.57E-298
**RSRP1**	4.81E-290	-0.6947236	0.489	0.704	1.52E-285
**KLRC1**	2.12E-285	-0.8288762	0.489	0.674	6.71E-281
**EIF3F**	5.11E-282	-0.5439784	0.697	0.834	1.62E-277
**HMGB2**	6.05E-278	-1.3320833	0.539	0.695	1.91E-273
**SUMO2**	1.63E-276	-0.5209904	0.738	0.864	5.17E-272
**PABPC1**	5.35E-270	-0.5133135	0.764	0.874	1.69E-265

Supplementary Table S2: Differentially expressed genes between cells from acute LCMV and MCMV-ie2-gp33. 50 most significant genes are shown.

	P_VAL	AVG_LOGFC	PCT.1	PCT.2	P_VAL_ADJ
**IFI27L2A**	0	2.71543905	0.902	0.376	0
**TOX**	0	1.97363858	0.561	0.04	0
**LAG3**	0	1.7982247	0.502	0.082	0
**PDCD1**	0	1.77186696	0.47	0.034	0
**BCL2**	0	1.5017254	0.522	0.106	0
**TIGIT**	0	1.41852719	0.425	0.063	0
**LY6A**	0	1.12476221	0.765	0.512	0
**VMP1**	0	1.10275855	0.526	0.207	0
**RTP4**	0	0.97442897	0.341	0.079	0
**GAPDH**	0	0.79228393	0.875	0.739	0
**S100A10**	0	-0.6637805	0.876	0.959	0
**HSPA8**	0	-0.7028137	0.834	0.916	0
**LY6C2**	0	-0.959187	0.591	0.935	0
**KLRK1**	0	-1.1480708	0.291	0.651	0
**CCR2**	0	-1.3439231	0.066	0.425	0
**KLRG1**	0	-1.6438252	0.128	0.579	0
**GABARAPL2**	4.96E-298	-0.9082782	0.554	0.76	1.57E-293
**OAS1A**	5.42E-297	0.75971003	0.25	0.042	1.71E-292
**CD8B1**	5.31E-296	-0.6709988	0.853	0.935	1.68E-291
**KLRD1**	9.50E-294	-0.8702225	0.459	0.765	3.01E-289
**TMSB10**	3.25E-293	-0.4140761	0.988	0.998	1.03E-288
**TAGLN2**	1.15E-279	-0.9379792	0.479	0.725	3.63E-275
**GZMA**	1.26E-279	-1.1646743	0.422	0.713	4.00E-275
**DNAJC15**	1.22E-274	-0.839141	0.462	0.715	3.86E-270
**BATF**	1.88E-267	1.05462086	0.441	0.177	5.93E-263
**AW112010**	1.31E-262	0.68208849	0.961	0.96	4.14E-258
**CCL3**	2.74E-253	1.49599451	0.323	0.097	8.66E-249
**ISG15**	1.33E-240	1.18920146	0.358	0.124	4.19E-236
**RSRP1**	1.03E-220	-0.7946892	0.494	0.704	3.25E-216
**THY1**	2.34E-220	-0.5313913	0.854	0.936	7.41E-216
**IL7R**	2.89E-220	-1.3104989	0.117	0.37	9.15E-216
**EPSTI1**	1.24E-217	-0.7301399	0.615	0.769	3.91E-213
**S1PR5**	5.43E-217	-0.944995	0.166	0.448	1.72E-212
**ARHGDIB**	1.86E-215	-0.456606	0.903	0.966	5.90E-211
**GM11808**	8.13E-212	0.67733389	0.807	0.641	2.57E-207
**SGK1**	7.14E-207	-0.9414159	0.149	0.406	2.26E-202
**PSMB8**	1.66E-201	0.54669681	0.901	0.835	5.26E-197
**INPP4B**	1.72E-196	0.76423166	0.294	0.096	5.43E-192
**KRTCAP2**	9.30E-192	-0.6344886	0.636	0.783	2.94E-187
**TSC22D3**	5.51E-185	-0.9219078	0.18	0.42	1.74E-180
**PGLYRP1**	2.35E-181	0.75060148	0.542	0.31	7.43E-177
**MALAT1**	2.40E-176	0.59084849	0.997	0.997	7.58E-172
**HNRNPF**	1.42E-173	-0.5668957	0.702	0.829	4.48E-169
**ADAM19**	6.86E-172	0.81056136	0.308	0.118	2.17E-167
**CD3G**	6.33E-165	0.58013492	0.928	0.899	2.00E-160
**GRAMD3**	3.41E-163	-0.794832	0.226	0.452	1.08E-158
**AHNAK**	3.57E-151	-0.5476839	0.639	0.809	1.13E-146
**ZEB2**	4.28E-151	-0.6879402	0.278	0.518	1.36E-146
**SH2D2A**	1.91E-147	0.84245867	0.56	0.371	6.05E-143

Supplementary Table S3: Differentially expressed genes between cells from chronic LCMV and MCMV-ie2-gp33. 50 most significant genes are shown.

	pathway	pval	padj	log2err	ES	NES	size
**1**	GSE13547_CTRL_VS_ANTI_IGM_STIM_BCELL_2H_DN	1.00E-10	2.30E-07	NA	0.90561224	3.99925697	18
**2**	GSE13547_CTRL_VS_ANTI_IGM_STIM_BCELL_12H_DN	1.00E-10	2.30E-07	NA	0.84914308	3.74988456	18
**3**	GSE9650_EXHAUSTED_VS_MEMORY_CD8_TCELL_DN	1.61E-10	2.47E-07	0.8266573	0.71928524	3.62693574	23
**4**	GSE39110_DAY3_VS_DAY6_POST_IMMUNIZATION_CD8_TCELL_WITH_IL2_TREATMENT_DN	1.44E-07	0.00016548	0.69013246	0.77535501	3.09092997	14
**5**	GSE39110_DAY3_VS_DAY6_POST_IMMUNIZATION_CD8_TCELL_UP	5.81E-07	0.0003339	0.6594444	0.6569128	3.02823487	20
**6**	GSE30962_ACUTE_VS_CHRONIC_LCMV_SECONDARY_INF_CD8_TCELL_UP	7.14E-06	0.00252303	0.61052688	0.68379461	2.81723083	15
**7**	GSE36888_STAT5_AB_KNOCKIN_VS_WT_TCELL_IL2_TREATED_17H_UP	1.48E-05	0.00323478	0.59332548	0.66724458	2.74904478	15
**8**	GSE13547_WT_VS_ZFX_KO_BCELL_ANTI_IGM_STIM_2H_UP	3.56E-05	0.00640414	0.55733224	0.67535741	2.69229245	14
**9**	GSE14415_INDUCED_TREG_VS_FOXP3_KO_INDUCED_TREG_UP	4.64E-06	0.00193716	0.61052688	0.77967497	2.66130577	10
**10**	GSE40068_CXCR5POS_BCL6POS_TFH_VS_CXCR5NEG_BCL6NEG_CD4_TCELL_UP	5.16E-06	0.00197675	0.61052688	0.77686271	2.65170652	10
**11**	GSE5142_CTRL_VS_HTERT_TRANSDUCED_CD8_TCELL_EARLY_PASSAGE_CLONE_DN	1.08E-05	0.00292392	0.59332548	0.80299252	2.62436326	9
**12**	GSE15330_HSC_VS_MEGAKARYOCYTE_ERYTHROID_PROGENITOR_UP	1.62E-06	0.00082876	0.64355184	0.88772301	2.59346657	7
**13**	GSE13522_CTRL_VS_T_CRUZI_Y_STRAIN_INF_SKIN_IFNAR_KO_UP	3.85E-05	0.00655737	0.55733224	0.69472684	2.59257834	12
**14**	GSE13547_WT_VS_ZFX_KO_BCELL_ANTI_IGM_STIM_12H_DN	6.80E-05	0.00935417	0.5384341	0.62461461	2.57340949	15
**15**	GSE7852_TREG_VS_TCONV_LN_DN	1.28E-05	0.00309041	0.59332548	0.83602661	2.56711385	8

Supplementary Table S4: Top 15 gene sets based on the C7 immunological signatures from the Broad institute. The upregulated genes from acute LCMV versus chronic LCMV infection were supplied as input.

	pathway	pval	padj	log2err	ES	NES	size
1	GSE3720_UNSTIM_VS_LPS_STIM_VD2_GAMMADELTA_TCELL_UP	5.89E-07	0.00020774	0.6594444	0.73314284	2.98372745	13
2	GSE9650_EFFECTOR_VS_MEMORY_CD8_TCELL_DN	6.97E-07	0.00022846	0.6594444	0.80776423	2.88401146	10
3	KAECH_DAY15_EFF_VS_MEMORY_CD8_TCELL_DN	4.54E-06	0.00086759	0.61052688	0.72371902	2.72162485	11
4	GSE13547_CTRL_VS_ANTI_IGM_STIM_BCELL_12H_DN	1.49E-05	0.00213407	0.59332548	0.66649112	2.71246982	13
5	GSE3565_CTRL_VS_LPS_INJECTED_SPLENOCYTES_DN	4.95E-05	0.00540343	0.55733224	0.64181174	2.61203027	13
6	KAECH_DAY8_EFF_VS_MEMORY_CD8_TCELL_DN	2.21E-05	0.00290112	0.57561026	0.76188164	2.57572169	9
7	GSE13547_CTRL_VS_ANTI_IGM_STIM_BCELL_2H_DN	0.00010062	0.00824395	0.5384341	0.57207517	2.51076206	15
8	GSE36888_STAT5_AB_KNOCKIN_VS_WT_TCELL_IL2_TREATED_17H_UP	9.60E-05	0.00800999	0.5384341	0.69462547	2.48006503	10
9	GSE39110_DAY3_VS_DAY6_POST_IMMUNIZATION_CD8_TCELL_UP	0.00022375	0.01571429	0.51884808	0.60258052	2.45236798	13
10	GSE14415_INDUCED_TREG_VS_FOXP3_KO_INDUCED_TREG_UP	5.34E-05	0.00556544	0.55733224	0.84701109	2.39324672	6
11	GOLDRATH_EFF_VS_MEMORY_CD8_TCELL_DN	0.00012749	0.01008478	0.51884808	0.70430305	2.3810636	9
12	GSE3920_IFNA_VS_IFNG_TREATED_FIBROBLAST_DN	1.47E-05	0.00213407	0.59332548	0.91079812	2.35379236	5
13	GSE33162_UNTREATED_VS_4H_LPS_STIM_HDAC3_HET_MACROPHAGE_DN	1.61E-05	0.00223669	0.57561026	0.90963929	2.35079756	5
14	GSE3720_LPS_VS_PMA_STIM_VD1_GAMMADELTA_TCELL_DN	0.00054618	0.02880326	0.47727082	0.5716097	2.32632366	13
15	GSE9650_NAIVE_VS_EFF_CD8_TCELL_UP	0.00034161	0.02062233	0.49849311	0.71402796	2.28543791	8

Supplementary Table S5: Top 15 gene sets based on the C7 immunological signatures from the Broad institute. The upregulated genes from acute LCMV versus MCMV-ie2-gp33 infection were supplied as input.

	pathway	pval	padj	log2err	ES	NES	size
1	GSE7219_WT_VS_NIK_NFKB2_KO_DC_UP	2.71E-07	0.00039659	0.67496286	0.71392853	2.64660834	21
2	GSE13547_CTRL_VS_ANTI_IGM_STIM_BCELL_2H_UP	4.75E-07	0.00052149	0.67496286	0.7917651	2.63802245	14
3	GSE14386_UNTREATED_VS_IFNA_TREATED_ACT_PBMC_MS_PATIENT_DN	1.20E-06	0.00105405	0.64355184	0.77614101	2.49931263	13
4	GSE7219_UNSTIM_VS_LPS_AND_ANTI_CD40_STIM_NIK_NFKB2_KO_DC_DN	1.08E-05	0.00684309	0.59332548	0.6626295	2.43994139	20
5	GSE13484_UNSTIM_VS_YF17D_VACCINE_STIM_PBMC_DN	1.20E-05	0.00684309	0.59332548	0.68937017	2.38144837	17
6	GSE10325_CD4_TCELL_VS_LUPUS_CD4_TCELL_DN	5.29E-05	0.01785321	0.55733224	0.68801126	2.35792476	16
7	GSE30962_ACUTE_VS_CHRONIC_LCMV_PRIMARY_INF_CD8_TCELL_DN	2.22E-05	0.00886642	0.57561026	0.77172159	2.35219299	11
8	GSE14415_INDUCED_TREG_VS_FAILED_INDUCED_TREG_DN	8.64E-05	0.02107168	0.5384341	0.62411979	2.29814054	20
9	GSE42021_TREG_VS_TCONV_PLN_UP	0.00024582	0.03480322	0.49849311	0.67877022	2.26154331	14
10	GSE2706_UNSTIM_VS_8H_LPS_DC_DN	4.95E-05	0.01785321	0.55733224	0.80194015	2.25947498	9
11	GSE6259_DEC205_POS_DC_VS_BCELL_DN	0.00011108	0.02437679	0.5384341	0.71781997	2.25730569	12
12	GSE43863_TFH_VS_LY6C_LOW_CXCR5NEG_EFFECTOR_CD4_TCELL_UP	0.00020271	0.03295088	0.51884808	0.66731486	2.25084058	15
13	GSE6269_HEALTHY_VS_STAPH_AUREUS_INF_PBMC_UP	6.79E-05	0.01879543	0.5384341	0.79597532	2.242669	9
14	GSE3982_DC_VS_TH1_DN	6.79E-05	0.01879543	0.5384341	0.7956327	2.24170367	9
15	GSE13485_CTRL_VS_DAY7_YF17D_VACCINE_PBMC_DN	0.00025591	0.03510022	0.49849311	0.62407886	2.19997712	18

Supplementary Table S6: Top 15 gene sets based on the C7 immunological signatures from the Broad institute. The upregulated genes from acute LCMV versus MCMV-ie2-gp33 infection were supplied as input.

	P_VAL	AVG_LOG2FC	PCT.1	PCT.2	P_VAL_ADJ
**GZMK**	2.81E-209	-1.9060771	0.134	0.597	8.88E-205
**UBA52**	6.32E-90	0.58079804	0.938	0.842	2.00E-85
**CCL5**	6.12E-89	-0.3131266	1	1	1.94E-84
**KLRB1C**	4.21E-73	0.99692946	0.304	0.097	1.33E-68
**GZMA**	1.69E-71	-0.967432	0.529	0.737	5.35E-67
**S100A4**	2.36E-70	0.87984878	0.616	0.392	7.45E-66
**H2-K1**	4.29E-69	-0.3651277	0.985	0.996	1.36E-64
**RSRP1**	1.79E-65	-0.805499	0.384	0.628	5.67E-61
**HSPA8**	5.31E-57	-0.5930809	0.714	0.854	1.68E-52
**EIF3F**	2.49E-49	-0.5849863	0.538	0.729	7.89E-45
**LARS2**	9.50E-47	0.723629	0.787	0.642	3.01E-42
**LY6C2**	9.51E-47	0.4008867	0.967	0.939	3.01E-42
**H2-D1**	4.07E-42	-0.2830918	0.98	0.995	1.29E-37
**EEF2**	6.71E-42	-0.4140489	0.809	0.906	2.12E-37
**PABPC1**	1.55E-40	-0.4942008	0.659	0.804	4.90E-36
**NKG7**	7.45E-40	-0.2863752	0.985	0.995	2.36E-35
**GM42418**	2.21E-38	0.55506498	0.981	0.963	6.98E-34
**IFI27L2A**	2.35E-37	-1.0121015	0.095	0.244	7.45E-33
**GM11808**	4.77E-37	0.58425695	0.634	0.513	1.51E-32
**ITGA4**	2.85E-34	-0.5353152	0.494	0.66	9.02E-30
**SERPINA3G**	3.80E-34	-0.5995364	0.011	0.105	1.20E-29
**H2-Q4**	4.47E-33	-0.5793434	0.307	0.493	1.41E-28
**GAPDH**	4.01E-31	0.45288926	0.706	0.592	1.27E-26
**SATB1**	1.11E-28	0.59957172	0.141	0.048	3.51E-24
**S1PR5**	7.23E-28	-0.6112009	0.319	0.474	2.29E-23
**SARAF**	4.22E-27	-0.5125861	0.314	0.48	1.33E-22
**RBM3**	1.96E-26	0.35903279	0.765	0.672	6.19E-22
**GRCC10**	3.68E-25	0.4439036	0.641	0.54	1.16E-20
**ZDHHC20**	1.65E-24	-0.5143226	0.183	0.324	5.21E-20
**NME2**	7.30E-24	0.32505862	0.812	0.746	2.31E-19
**TNFRSF25**	2.10E-23	0.51177342	0.139	0.054	6.64E-19
**CD3E**	4.82E-23	-0.2609217	0.892	0.938	1.53E-18
**MYL6**	8.37E-23	0.25142513	0.952	0.94	2.65E-18
**AW112010**	8.98E-23	-0.2757757	0.899	0.95	2.84E-18
**SP100**	1.87E-22	-0.3974515	0.542	0.681	5.91E-18
**IL7R**	3.60E-22	0.48484611	0.423	0.289	1.14E-17
**FTH1**	4.27E-22	0.26867576	0.951	0.935	1.35E-17
**LAPTM5**	6.03E-22	-0.2657018	0.891	0.945	1.91E-17
**REEP5**	2.50E-21	-0.4382126	0.456	0.595	7.91E-17
**JUNB**	6.33E-21	-0.4571102	0.381	0.535	2.00E-16
**PYCARD**	9.58E-21	-0.4616674	0.312	0.456	3.03E-16
**SGK1**	9.78E-21	-0.5092986	0.212	0.343	3.09E-16
**TSC22D3**	3.44E-20	-0.511426	0.24	0.371	1.09E-15
**IER3**	2.25E-19	0.49437024	0.133	0.057	7.12E-15
**CTSS**	4.06E-19	-0.4639295	0.081	0.177	1.28E-14
**DNAJC15**	9.59E-19	0.34835993	0.694	0.632	3.03E-14
**CHD3**	1.23E-18	-0.4524719	0.211	0.334	3.88E-14
**SH3BGRL3**	1.56E-18	0.25396395	0.916	0.912	4.95E-14
**CD48**	2.09E-18	-0.3561818	0.581	0.701	6.62E-14

Supplementary Table S7: Differentially expressed genes between cells from acute LCMV and MCMV-ie2-gp33 infections in shared transcriptional cluster 1. 50 most significant genes are shown

	P_VAL	AVG_LOG2FC	PCT.1	PCT.2	P_VAL_ADJ
**IFI27L2A**	3.82E-70	1.90703918	0.71	0.244	1.21E-65
**BCL2**	3.41E-53	1.2522606	0.374	0.075	1.08E-48
**TOX**	2.22E-48	0.91048459	0.176	0.016	7.04E-44
**CCL5**	6.72E-45	0.44169179	1	1	2.13E-40
**S100A4**	1.02E-40	1.17603126	0.744	0.392	3.21E-36
**ST6GALNAC3**	1.10E-35	0.63125518	0.111	0.008	3.47E-31
**KLRA9**	5.36E-33	1.42237246	0.225	0.044	1.70E-28
**KLRB1C**	6.81E-27	0.94111074	0.324	0.097	2.15E-22
**LTB**	2.62E-26	-1.6334296	0.095	0.42	8.30E-22
**LY6A**	4.46E-26	0.94991917	0.58	0.281	1.41E-21
**BCL2L11**	9.73E-26	0.76274004	0.141	0.022	3.08E-21
**RSRP1**	2.52E-24	-1.096399	0.355	0.628	7.96E-20
**S100A6**	3.22E-23	0.78028039	0.958	0.881	1.02E-18
**KLRE1**	4.98E-22	0.85996068	0.485	0.21	1.58E-17
**CD7**	6.00E-21	-1.4194125	0.069	0.351	1.90E-16
**LAT**	1.03E-20	-0.7206973	0.664	0.809	3.25E-16
**GZMK**	3.31E-20	-1.0539035	0.302	0.597	1.05E-15
**IL7R**	2.65E-19	-1.635044	0.031	0.289	8.39E-15
**GZMB**	2.61E-18	0.81107646	0.687	0.431	8.26E-14
**HSPA8**	2.38E-16	-0.6467477	0.775	0.854	7.53E-12
**CORO1A**	1.49E-15	-0.5060116	0.817	0.912	4.71E-11
**GM11808**	3.16E-15	0.62345235	0.74	0.513	9.99E-11
**HSP90AB1**	8.70E-15	-0.5932951	0.794	0.856	2.75E-10
**BHLHE40**	4.31E-14	0.75482499	0.385	0.187	1.36E-09
**RBM3**	2.64E-13	0.49531071	0.832	0.672	8.37E-09
**HNRNPF**	2.11E-12	-0.5416678	0.626	0.749	6.67E-08
**GM10522**	9.37E-12	0.49973061	0.141	0.043	2.96E-07
**KLRG1**	1.17E-11	-0.7008433	0.351	0.55	3.71E-07
**KLRC1**	1.51E-11	0.53817703	0.821	0.684	4.77E-07
**LAG3**	2.05E-11	0.54020208	0.145	0.046	6.47E-07
**CCR2**	3.27E-11	-0.8982728	0.107	0.297	1.03E-06
**RTP4**	3.93E-11	0.51476013	0.126	0.037	1.24E-06
**AW112010**	6.19E-11	0.38316637	0.981	0.95	1.96E-06
**GABARAPL2**	8.51E-10	-0.5114362	0.595	0.714	2.69E-05
**GEM**	1.20E-09	0.50377405	0.107	0.032	3.78E-05
**ACTG1**	1.97E-09	-0.378246	0.897	0.939	6.23E-05
**CTLA2A**	2.70E-09	-0.7922477	0.153	0.325	8.53E-05
**LCP1**	6.47E-09	-0.6235451	0.405	0.544	0.00020456
**RAP1B**	9.77E-09	0.53591787	0.584	0.403	0.00030903
**SARAF**	1.03E-08	-0.5791809	0.355	0.48	0.00032471
**TAGLN2**	1.24E-08	-0.5765322	0.477	0.598	0.00039117
**PLEK**	1.80E-08	0.54637102	0.282	0.146	0.00056969
**LGALS3**	2.99E-08	0.61040523	0.519	0.349	0.00094443
**LARS2**	4.82E-08	0.36691488	0.813	0.642	0.00152332
**MALAT1**	8.12E-08	0.28716038	1	0.999	0.00256952
**EIF1**	8.46E-08	-0.2794213	0.939	0.972	0.00267738
**ARPC1B**	1.07E-07	0.28981923	0.908	0.823	0.0033889
**GAPDH**	1.08E-07	0.45576962	0.767	0.592	0.00341654
**XAF1**	1.50E-07	0.40432911	0.16	0.067	0.00475109

Supplementary Table S8: Differentially expressed genes between cells from chronic LCMV and MCMV-ie2-gp33 infections in shared transcriptional cluster 1. 50 most significant genes are shown.

	P_VAL	AVG_LOGFC	PCT.1	PCT.2	P_VAL_ADJ
**DOCK10**	1.34E-06	-0.2882731	0.293	0.484	0.04224378
**ATF7IP**	7.05E-06	-0.2523825	0.179	0.312	0.22303645
**ANP32B**	8.49E-06	-0.2552188	0.208	0.351	0.26864323
**PHIP**	1.76E-05	-0.2732882	0.13	0.24	0.55701344
**SETD2**	2.61E-05	-0.2821978	0.085	0.172	0.82536605
**IFI27L2A**	4.23E-05	-0.3957353	0.222	0.351	1
**MYH9**	0.000146	-0.2576595	0.623	0.756	1
**ZMYND8**	0.00014752	-0.253812	0.055	0.122	1
**XIST**	0.00040271	-0.3019917	0.585	0.746	1
**HOPX**	0.00062951	0.35250665	0.628	0.616	1
**CAPZB**	0.00333798	0.25674124	0.692	0.724	1
**KANSL1**	0.00490765	-0.2803346	0.129	0.197	1
**HMOX2**	0.02608445	0.35056952	0.212	0.168	1
**KLRC1**	0.07362607	0.28927889	0.553	0.556	1
**FIBP**	0.10582023	0.25601526	0.189	0.158	1
**KLRE1**	0.19862523	0.25666786	0.066	0.047	1
**KCNJ8**	0.2308335	0.39613536	0.18	0.165	1
**GM45552**	0.26191045	0.27161242	0.271	0.262	1
**PDIA3**	0.29045391	0.2543376	0.45	0.487	1
**FAM204A**	0.3211637	0.29126211	0.284	0.29	1
**GZMA**	0.35304194	1.14922248	0.2	0.186	1
**HBB-BS**	0.35319487	0.60579736	0.006	0.011	1
**ANXA1**	0.35431144	0.31233606	0.156	0.143	1
**ZGPAT**	0.53789233	0.25344794	0.19	0.194	1
**CX3CR1**	0.54860923	0.41123232	0.225	0.233	1
**CCL4**	0.60412083	0.32413914	0.323	0.348	1
**KLRG1**	0.71538448	0.29976692	0.27	0.29	1

Supplementary Table S9: Differentially expressed genes between expanded (>1 cell) and lowly expanded (1 cell) clones from acute LCMV infection.

	P_VAL	AVG_LOGFC	PCT.1	PCT.2	P_VAL_ADJ
**CCR2**	5.26E-12	-0.6316694	0.048	0.212	1.66E-07
**NKG7**	2.81E-10	0.60104259	0.988	0.923	8.89E-06
**SELL**	1.48E-09	-0.6637067	0.016	0.106	4.68E-05
**XYLT2**	2.13E-08	-0.2719654	0.012	0.087	0.00067375
**CAPG**	4.66E-08	-0.8660152	0.062	0.202	0.00147501
**CCL5**	5.71E-08	0.63381807	0.949	0.846	0.00180655
**KLRG1**	6.39E-07	-0.993421	0.101	0.25	0.02021717
**LY6C2**	1.75E-06	-0.9227214	0.586	0.702	0.05534647
**FOXP3**	2.70E-06	-0.3241355	0.003	0.038	0.08530875
**ASS1**	2.70E-06	-0.4686685	0.066	0.192	0.08541885
**TMSB10**	5.83E-05	-0.277983	0.997	0.99	1
**ATP6V1G1**	9.41E-05	-0.358435	0.384	0.587	1
**TSPAN14**	0.00010208	-0.3081298	0.091	0.212	1
**PDLIM1**	0.00011084	-0.3530901	0.095	0.212	1
**UGCG**	0.0001499	-0.2671773	0.085	0.202	1
**PDCD1**	0.00016519	0.62672739	0.514	0.317	1
**EMB**	0.0001957	-0.4902165	0.164	0.298	1
**FYN**	0.0002149	0.59914093	0.498	0.337	1
**DAPL1**	0.00022667	-0.3972859	0.012	0.058	1
**TOX**	0.00023073	0.53032574	0.625	0.481	1
**KREMEN1**	0.00025029	-0.2558287	0.003	0.029	1
**PSMC4**	0.00026775	-0.3892143	0.223	0.385	1
**SATB1**	0.00026939	-0.3391468	0.044	0.125	1
**TCP1**	0.00028258	-0.4158665	0.314	0.471	1
**TPD52L2**	0.00028508	-0.3674921	0.146	0.279	1
**CLEC2I**	0.00028954	-0.4777998	0.16	0.298	1
**PUS1**	0.00029749	-0.2737443	0.061	0.154	1
**SH2D2A**	0.00032462	0.57682721	0.594	0.471	1
**COL16A1**	0.00033644	-0.5504194	0	0.01	1
**IZUMO1R**	0.00056926	-0.4234173	0.064	0.154	1
**TTC9C**	0.00075394	-0.4085322	0.094	0.192	1
**ETNK1**	0.00090396	-0.2782523	0.078	0.173	1
**CD3E**	0.00100254	0.25312934	0.988	0.981	1
**ZFP672**	0.00104327	-0.2721226	0.091	0.192	1
**CTSS**	0.00118435	-0.4875581	0.269	0.394	1
**LY6E**	0.00120367	-0.6356878	0.706	0.808	1
**FRA10AC1**	0.00123056	-0.3090862	0.081	0.173	1
**KLRK1**	0.00134651	-0.5068856	0.265	0.404	1
**TECPR1**	0.00137483	-0.3922136	0.152	0.269	1
**IL7R**	0.00139261	-0.6661117	0.103	0.202	1
**NSG2**	0.0015329	-0.268503	0.069	0.154	1
**RINL**	0.0016614	0.43408775	0.597	0.462	1
**PPP1R35**	0.00181868	-0.2780857	0.13	0.24	1
**PEPD**	0.00183022	-0.4057506	0.058	0.135	1
**GBP5**	0.00199544	-0.2554559	0.052	0.125	1
**TRIP12**	0.002115	0.51826498	0.263	0.135	1
**IGFLR1**	0.00215676	-0.3257925	0.016	0.058	1
**LAG3**	0.0021933	0.63604649	0.495	0.365	1
**TRAF2**	0.00219821	-0.2895937	0.104	0.202	1

Supplementary Table S10: Differentially expressed genes between expanded (>1 cell) and lowlyd (1 cell) clones from chronic LCMV infection. 50 most significant genes are shown.

	P_VAL	AVG_LOGFC	PCT.1	PCT.2	P_VAL_ADJ
**SELL**	1.92E-38	-1.1977319	0.031	0.283	6.08E-34
**FOXP3**	2.89E-33	-0.5390594	0.001	0.088	9.14E-29
**RAMP1**	3.03E-29	-0.4107768	0.001	0.08	9.60E-25
**IZUMO1R**	7.33E-27	-1.1483387	0.004	0.097	2.32E-22
**TNFRSF4**	6.12E-25	-1.4680197	0.003	0.088	1.94E-20
**IGFBP4**	1.87E-21	-0.3971582	0.001	0.062	5.91E-17
**ATP1B1**	1.89E-21	-0.3173698	0.001	0.062	5.99E-17
**LGALS1**	3.06E-21	1.14257112	0.963	0.796	9.68E-17
**PACSIN1**	1.03E-20	-0.5167923	0.014	0.142	3.27E-16
**RGS10**	6.92E-19	-0.6966717	0.023	0.168	2.19E-14
**S100A6**	2.21E-16	0.95443677	0.956	0.726	6.99E-12
**CD83**	7.80E-16	-0.4167907	0.002	0.053	2.47E-11
**CCR7**	2.30E-15	-0.6641685	0.031	0.177	7.28E-11
**NKG7**	2.03E-14	0.61159956	0.996	0.876	6.42E-10
**NSG2**	2.51E-14	-0.5583056	0.027	0.159	7.94E-10
**PENK**	3.02E-14	-0.6112972	0	0.027	9.54E-10
**IKZF2**	1.05E-13	-0.8268305	0.007	0.08	3.33E-09
**S100A10**	1.55E-13	0.62169771	0.981	0.947	4.89E-09
**CCL5**	1.64E-13	0.6845331	0.999	0.841	5.20E-09
**DAPL1**	2.33E-13	-0.7568471	0.021	0.133	7.38E-09
**ID2**	3.63E-13	0.86762081	0.84	0.558	1.15E-08
**ID3**	9.18E-13	-0.824152	0.025	0.142	2.91E-08
**CRIP1**	4.28E-12	0.67648638	0.982	0.929	1.35E-07
**GZMA**	1.10E-11	0.76075899	0.797	0.478	3.49E-07
**S100A4**	1.92E-11	1.03273282	0.663	0.381	6.07E-07
**SLFN5**	2.79E-11	-0.438128	0.018	0.115	8.81E-07
**TCF7**	1.10E-10	-0.7415965	0.193	0.434	3.48E-06
**EEF1B2**	1.29E-10	-0.6179274	0.894	0.965	4.07E-06
**KLRG1**	2.87E-10	0.78522652	0.686	0.372	9.07E-06
**GABARAPL2**	2.94E-10	0.72035881	0.833	0.655	9.31E-06
**LGALS3**	3.05E-10	1.04977271	0.627	0.345	9.65E-06
**CYTH3**	3.29E-10	-0.2644768	0.004	0.053	1.04E-05
**LY6E**	5.03E-10	-0.7835596	0.653	0.876	1.59E-05
**REEP5**	5.33E-10	0.77003679	0.779	0.513	1.69E-05
**LIMD2**	6.63E-10	-0.6387403	0.65	0.832	2.10E-05
**GZMB**	8.57E-10	0.87845043	0.673	0.381	2.71E-05
**VIM**	2.03E-09	0.91755663	0.856	0.717	6.41E-05
**LTA**	2.36E-09	-0.3640842	0.009	0.071	7.48E-05
**CTSD**	7.32E-09	0.57677463	0.879	0.717	0.00023164
**TPT1**	8.02E-09	-0.310855	0.999	0.991	0.00025366
**IL6RA**	1.53E-08	-0.4451933	0.015	0.088	0.00048307
**KLRC1**	1.66E-08	0.59938241	0.797	0.496	0.00052511
**INPP4B**	1.95E-08	-0.6986722	0.103	0.265	0.00061586
**LTB**	1.95E-08	-0.7952277	0.587	0.796	0.00061607
**EPHX1**	2.63E-08	-0.3471904	0.018	0.097	0.00083239
**CTLA4**	3.52E-08	-0.3421417	0.013	0.08	0.00111269
**GZMK**	3.88E-08	0.67618973	0.785	0.531	0.00122604
**JAK1**	5.84E-08	0.59140543	0.815	0.593	0.00184865
**ABRACL**	6.08E-08	0.58082327	0.835	0.681	0.00192226

Supplementary Table S11: Differentially expressed genes between expanded (>1 cell) and lowly expanded (1 cell) clones from MCMV-ie2-gp33 infection. 50 most significant genes are shown.
